# Phosphatidylinositol-3,4,5-trisphosphate interacts with alpha-synuclein and initiates its aggregation and formation of Parkinson’s disease-related fibril polymorphism

**DOI:** 10.1007/s00401-023-02555-3

**Published:** 2023-03-20

**Authors:** Chi-Jing Choong, César Aguirre, Keita Kakuda, Goichi Beck, Hiroki Nakanishi, Yasuyoshi Kimura, Shuichi Shimma, Kei Nabekura, Makoto Hideshima, Junko Doi, Keiichi Yamaguchi, Kichitaro Nakajima, Tomoya Wadayama, Hideki Hayakawa, Kousuke Baba, Kotaro Ogawa, Toshihide Takeuchi, Shaymaa Mohamed Mohamed Badawy, Shigeo Murayama, Seiichi Nagano, Yuji Goto, Yohei Miyanoiri, Yoshitaka Nagai, Hideki Mochizuki, Kensuke Ikenaka

**Affiliations:** 1 Department of Neurology, Osaka University Graduate School of Medicine, 2-2 Yamadaoka, Suita, Osaka 565-0871 Japan; 2 Lipidome Lab Co., Ltd, Akita-City, Akita 010-0825 Japan; 3 Department of Biotechnology, Graduate School of Engineering, Osaka University, 2-1 Yamadaoka, Suita, Osaka 565-0871 Japan; 4 Global Center for Medical Engineering and Informatics, Osaka University, 2-1 Yamadaoka, Suita, Osaka 565-0871 Japan; 5 Department of Neurology, Kindai University, 3-4-1 Kowakae, Higashiosaka City, Osaka 577-8502 Japan; 6 Department of Agricultural Biochemistry, Faculty of Agriculture, Zagazig University, Zagazig, Egypt; 7 Brain Bank for Neurodevelopmental, Neurological and Psychiatric Disorders, United Graduate School of Child Development, Osaka University, 2-2 Yamadaoka, Suita, Osaka 565-0871 Japan; 8 Institute for Protein Research, Osaka University, 3-2 Yamadaoka, Suita, Osaka 565-0871 Japan

**Keywords:** Alpha-synuclein, Synaptojanin 1, Phosphatidylinositol-3,4,5-trisphosphate, Parkinson’s disease

## Abstract

Lipid interaction with α-synuclein (αSyn) has been long implicated in the pathogenesis of Parkinson’s disease (PD). However, it has not been fully determined which lipids are involved in the initiation of αSyn aggregation in PD. Here exploiting genetic understanding associating the loss-of-function mutation in Synaptojanin 1 (SYNJ1), a phosphoinositide phosphatase, with familial PD and analysis of postmortem PD brains, we identified a novel lipid molecule involved in the toxic conversion of αSyn and its relation to PD. We first established a SYNJ1 knockout cell model and found SYNJ1 depletion increases the accumulation of pathological αSyn. Lipidomic analysis revealed SYNJ1 depletion elevates the level of its substrate phosphatidylinositol-3,4,5-trisphosphate (PIP_3_). We then employed *Caenorhabditis elegans* model to examine the effect of SYNJ1 defect on the neurotoxicity of αSyn. Mutations in SYNJ1 accelerated the accumulation of αSyn aggregation and induced locomotory defects in the nematodes. These results indicate that functional loss of SYNJ1 promotes the pathological aggregation of αSyn via the dysregulation of its substrate PIP_3_, leading to the aggravation of αSyn-mediated neurodegeneration. Treatment of cultured cell line and primary neurons with PIP_3_ itself or with PIP_3_ phosphatase inhibitor resulted in intracellular formation of αSyn inclusions. Indeed, in vitro protein–lipid overlay assay validated that phosphoinositides, especially PIP_3_, strongly interact with αSyn. Furthermore, the aggregation assay revealed that PIP_3_ not only accelerates the fibrillation of αSyn, but also induces the formation of fibrils sharing conformational and biochemical characteristics similar to the fibrils amplified from the brains of PD patients. Notably, the immunohistochemical and lipidomic analyses on postmortem brain of patients with sporadic PD showed increased PIP_3_ level and its colocalization with αSyn. Taken together, PIP_3_ dysregulation promotes the pathological aggregation of αSyn and increases the risk of developing PD, and PIP_3_ represents a potent target for intervention in PD.

**Supplementary Information:**

The online version contains supplementary material available at 10.1007/s00401-023-02555-3.

## Introduction

Parkinson’s disease (PD) is the second most common neurodegenerative disease after Alzheimer’s disease (AD), and its incidence is increasing with the aging of the global population. Clinically, PD is characterized by progressive motor and non-motor symptoms. Pathologically, its hallmarks include progressive neuronal loss mainly involving dopaminergic neurons in the substantia nigra and the appearance of neuronal inclusions called Lewy bodies (LB), which are predominantly composed of aberrant α-synuclein (αSyn) aggregates [3, 4, 56]. Cumulative evidence has indicated that αSyn aggregation plays a significant role in the pathogenesis of both familial and sporadic forms of PD, and its cell-to-cell propagation is associated with disease progression [32, 42, 43]. Multiplication and missense mutations in the SNCA gene encoding αSyn that render it prone to aggregation are linked to familial forms of PD, but the initiating event of αSyn aggregation in sporadic PD remains unclear.

Many studies have examined the association between the interaction of αSyn with lipids, especially glucosylceramide, and the propensity of αSyn to aggregate [1, 2, 10, 22, 29, 37, 54, 57, 58, 63]. Glucosylceramide has been targeted because mutations in *glucocerebrosidase* (*GBA*) represent the single largest risk factor for the development of PD. These mutations cause a consequent loss of enzymatic activity and excessive buildup of the substrate glucosylceramide. Glucosylceramide converts physiological αSyn conformers into pathogenic species that are prone to aggregate formation [63]. Interestingly, in our previous study, structural analysis of Lewy bodies in the PD brain revealed that lipids were abundantly distributed in the core of Lewy bodies, even in idiopathic PD patients, indicating the involvement of some lipids in the initiation of αSyn aggregation [3].

Recently, a transcriptome-wide association study (TWAS) evaluating the protein–protein interaction network connectivity between protein products nominated by PD TWAS and monogenic genes unveiled the link between Synaptojanin 1 (SYNJ1) and SNCA [31]. SYNJ1 is a brain-enriched phosphatase that dephosphorylates phosphoinositides (PIPs) [36]. PIPs are a small group of cellular phospholipids composed of two fatty acid chains linked by a glycerol moiety to a water-soluble inositol head group. Phosphorylation at positions 3, 4, and 5 of the inositol rings of phosphatidylinositol yielded seven distinct phosphoinositide derivatives, PI-3-P, PI-4-P, PI-5-P, PI-3,4-P_2_, PI-3,5-P_2_, PI-4,5-P_2_ and PI-3,4,5-P_3_ (PIP_3_) [6]. SYNJ1 consists of an amino-terminal Sac1-like phosphatase domain that dephosphorylates PI-3-P and PI-4-P, a central inositol 5′-phosphatase domain that dephosphorylates PI-4,5-P_2_ and PIP_3_, and a carboxyl-terminal proline-rich region. Mutations identified in the two phosphatase domains of SYNJ1, R258Q, R459P and R839C, have been associated with autosomal recessive, early-onset familial type PD (PARK20), implying involvement of lipid dysregulation in PD pathogenesis [34, 41, 45]. Importantly, a study assessing genome-wide expression datasets for postmortem sporadic PD brains available in the public domain found that a subset of sporadic PD brains showed significant down-regulation of SYNJ1 transcript in various brain regions including the prefrontal cortex, striatum and substantia nigra [39].

By integrating genetic evidence, this study aimed to determine the lipid molecule involved in the physiological to pathological shift of αSyn in PD pathogenesis and the underlying mechanism. Towards this goal, we employed genetically engineered cellular and *Caenorhabditis elegans* (*C. elegans*) models and demonstrated that depletion of SYNJ1 is associated with higher intracellular αSyn accumulation, accompanied by locomotory defects in *C. elegans*. Interestingly, lipidomic analysis revealed SYNJ1 depletion elevates the level of its substrate PIP_3_. Cell-based assays showed that upregulation of intracellular PIP_3_ itself induces the formation of αSyn inclusions. Subsequent in vitro protein–lipid overlay and aggregation assays further confirmed PIP_3_ as a lipid molecule that can directly interact with αSyn monomer and initiate aggregation. Notably, PIP_3_ induces the formation of PD-like fibril polymorphism. Postmortem brain analysis also unveiled the involvement of PIP_3_ dysregulation in synucleinopathy of PD. Overall, our data demonstrate that the aberrant interaction of αSyn with PIP_3_ which accumulates upon the loss of SYNJ1 phosphatase activity, is a factor that prompts physiologic αsyn to misfold and form pathologic inclusions, thereby highlighting a substantial role of PIP_3_ in the context of PD pathogenesis.

## Materials and methods

### Cell culture

The human cervical adenocarcinoma cell line HeLa (ATCC^®^ CCL-2) and the human neuroblastoma cell line SH-SY5Y (ATCC^®^ CRL-2266) were cultured in Dulbecco’s modified Eagle’s medium (Sigma-Aldrich, D5796) supplemented with 10% fetal bovine serum, 100 U/mL penicillin, and 100 μg/mL streptomycin at 37 °C in a 95% air and 5% CO_2_ humidified incubator. Cells were routinely passaged after reaching 75–90% confluency.

### Plasmid and virus production

The expression vectors for αSyn-GFP and αSyn-mRFP were created using the Multisite Gateway system, according to the manufacturer’s protocol (12537100, Invitrogen). Briefly, complementary DNA (cDNA) of αSyn with a stop codon was inserted between the attL1 and attR5 sites of the entry vector. Then, this was recombined with pENTR-L5-EGFP-L2 or pENTR-L5-mRFP-L2 (gift from Professor H. Kuroyanagi, University of the Ryukyus Graduate School of Medicine) into the destination vector with the CMV promoter pcDNA™-DEST40 (12274015, Invitrogen). To generate pMRX-ib-αSyn-mRFP for preparation of recombinant retroviruses, cDNA corresponding to αSyn-mRFP was subcloned into the pENTR1A plasmid. The sequences inserted into the pENTR1A plasmid were then transferred into pMRX-ib-DEST using Gateway LR Clonase. Recombinant retroviruses were prepared as previously described [46].

### gRNA design to target SYNJ1

Targeting sequences of sgRNA in the CRISPR/Cas9 system were determined using CRISPRdirect (https://crispr.dbcls.jp/). The targeting sgRNA sequence used in this study was 5′-GGGACCAGGTTTAATGTCCG-3′. According to the Zhang Lab General cloning protocol (http://www.addgene.org/crispr/zhang/), synthesized and annealed sgRNA targeting human *SYNJ1* was inserted into the modified pX330 plasmid (pX330; Addgene #42230) in which a P2A-puromycin resistance gene was conjugated to Cas9 (hereafter referred to as pCas9-puroR).

### Establishment of SYNJ1-KO stable SH-SY5Y cell line

SH-SY5Y cells were transfected with pCas9-puroR vectors, and the cells were selected using 1 µg/ml puromycin (P8833, Sigma-Aldrich) for 7 days. Single colonies were manually isolated under a microscope. Genomic DNA of isolated clones was extracted using the PureLink™ Genomic DNA Mini Kit (K1820-01, Invitrogen), and sequences were confirmed. Out of 16 clones, 5 clones contained the homozygous frameshift *SYNJ1* mutation. Depletion of SYNJ1 was confirmed by western blotting and immunostaining, and the established SYNJ1-KO lines were used for further experiments.

### Transfection by electroporation

For each electroporation reaction of SH-SY5Y, 1.5 × 10^6^ cells/100 μL optiMEM (Gibco, 31-985-062) was used. Ten μg of expression vectors encoding eGFP or αSyn-eGFP were added to the cell suspension. Cells/plasmid DNA suspensions were then transferred into 2-mm gap cuvettes (Nepa Gene Co., Ltd, EC-002S) and electroporated using a NEPA21 Super Electroporator (Nepa Gene Co., Ltd, Chiba, Japan). Immediately after electroporation, 500 μL of the pre-equilibrated culture medium was added to the cuvette, and the cell suspension was transferred to a 6-well plate. At 24 h post-transfection, media were replaced with fresh media; cells were harvested 72 h post-transfection for protein immunoblot analysis and immunostaining.

### Western blotting

Cells were harvested and lysed in CelLytic MT Cell Lysis Reagent (C2978, Sigma-Aldrich) with a protease inhibitor (539131, Calbiochem) and phosphatase inhibitor (07574-61, Nacalai Tesque) mixture. The protein concentration of cell lysates was determined using a Pierce™ BCA protein assay kit (23225, Thermo Scientific). Lysates (10 μg/lane) were resolved on 10–20% sodium dodecyl sulfate (SDS)–polyacrylamide gel (2331840, ATTO Corporation) and transferred to polyvinylidene difluoride membranes (1620177, Bio-Rad Laboratories, Inc.). The membranes were incubated with blocking buffer and probed with the following primary antibodies: anti-Synj1 (1:1000) (ATL ATLAS antibodies AB, HPA011916); anti-phosphorylated αSyn (1:1000) (014-20281, Wako); αSyn antibody (Syn211) (1:1000) (32-8100, Invitrogen) and ACTB/β-actin antibody, clone C4 (1:10,000) (MAB1501, Merck Millipore). Following incubation with Amersham ECL horseradish peroxidase-conjugated anti-rabbit or anti-mouse secondary antibody (1:20,000) (NA934, NA931, GE Healthcare), the bound antibody was visualized with Amersham ECL Prime western blotting Detection Reagent (RPN2236, GE Healthcare) using the ChemiDoc Touch Imaging System (BioRad, Berkeley, CA, USA).

### Immunocytochemistry

Cells were fixed with 4% PFA (09154-85, Nacalai Tesque) for 30 min at room temperature. After permeabilization with 0.1% Triton X-100 solution for 10 min and washing in tris-buffered saline (TBS), cells were blocked with 0.2% gelatin-TBS for 30 min and subsequently with primary antibodies, purified anti-PtdIns (3,4,5) P_3_ IgG (1:200) (Z-P345B, Echelon Biosciences), anti-Synj1 (1:500) (HPA011916, ATL ATLAS antibodies AB), anti-LAMP1 (1:100) (#9091, Cell Signaling Technology), and anti-LAMP-2 (sc-18822, Santa Cruz) overnight at 4 °C. After three washes in TBS, the cells were incubated with fluorescein AffiniPure donkey anti-mouse antibody (715-095-150, Jackson ImmunoResearch), Cy™3 AffiniPure donkey anti-Rabbit antibody (111-165-003, Jackson ImmunoResearch), or Alexa Fluor 647 donkey anti-rabbit antibody (A31573, Molecular Probes) (1:1000) for 1 h at room temperature. After washing in TBS, the cells were counterstained with Hoechst (H21486, Thermo Scientific) and observed microscopically using SpinSR10 (Olympus, Tokyo, Japan).

### LC–MS/MS analysis

Lipidome analysis was conducted according to the Lipidome lab Multiphospholipid Scan package (Lipidome lab, Akita, Japan), using liquid chromatography triple quadrupole mass spectrometry (LC–TQMS) based on the methods described previously [27, 53]. Fifty mg of cell pellets and brain samples were prepared and kept at − 80 ℃ until analysis. Briefly, the sample was dissolved with methanol, and then homogenized using a glass homogenizer. Total lipids were extracted using the liquid–liquid extraction as Bligh and Dyer methods [8]. In addition, to analyze acidic phospholipids (PLs) such as PI and PIP, another aliquot of the same lipid extract was added with an equal volume of methanol before being loaded onto a diethylaminoethyl-cellulose column (Santa Cruz Biotechnology) pre-equilibrated with chloroform. After successive washes with chloroform/methanol (1:1, v/v), the acidic PLs were eluted with chloroform/methanol/HCl/water (12:12:1:1, v/v). The resultant fraction was subjected to a methylation reaction with TMS-diazomethane, followed by evaporation to dryness to give a residue, which was re-dissolved in methanol.

LC–MS/MS analysis was performed using the Xevo TQ-XS mass spectrometer with an ACQUITY UPLC H-Class (Waters). The lipids were separated on a Waters X-Bridge C18 column (3.5 μm, 150 mm × 1.0 mm internal diameter) at 40 °C using a gradient solvent system as follows: mobile phase A was isopropanol/methanol/water (5/1/4 v/v/v) supplemented with 5 mM ammonium formate and 0.05% ammonium hydroxide (28% in water); mobile phase B was isopropanol supplemented with 5 mM ammonium formate and 0.05% ammonium hydroxide (28% in water) with flow rate was 80 μL/min. Lipid species were measured using multiple reaction monitoring (MRM) in positive ion mode. Peak areas of individual species were normalized with those of the internal/surrogate standards PI 15:0/18:1-d7, PI(4)P 17:0/20:4, PI(4,5)P_2_ 17:0/20:4 and PI(3,4,5)P_3_ 17:0/20:4 (Avanti Polar Lipids), which were added to the samples before lipid extraction. The LC–MS/MS raw data were processed using analytical software (MassLynx4.2; Waters).

### *Caenorhabditis elegans* culture and strains

Standard methods were used to culture *C. elegans* on nematode growth medium (NGM) agar seeded with OP50 Escherichia coli (*E. coli*) [9]. The worms were maintained at 20 °C unless otherwise indicated.

The following strains, obtained from the *C. elegans* Genetics Center, were used: N2 wild-type (Bristol), NL5901 pkIs2386 [unc-54p::alpha-synuclein::YFP] [20], and EG3027 (mutant unc-26:s1710) [47]. To create NL5901/N2 and NL5901/unc-26 heterozygote worms, NL5901 males were mated with N2 and unc-26 hermaphrodites, and F1 worms were used for the analysis. To create N2/N2 and N2/unc-26 heterozygote worms, N2 males were mated with N2 and unc-26 hermaphrodites.

### Quantification of αSyn aggregates formed in *C. elegans*

Aggregates were quantified as previously described, with some modifications [20, 23, 44]. Briefly, NL5901/N2 and NL5901/unc-26 heterozygote worms were created by mating. Synchronized nematodes were cultured until they reached the young adult stage (day 3). Animals were then transferred onto new NGM plates and cultured until adult day 2 stage, and αSyn aggregates were counted under a Zeiss LSM 700 confocal microscope. For each independent experiment, 14 worms from each group were examined. Aggregates were defined as discrete bright structures with boundaries distinguishable from the surrounding fluorescence. The aggregates were measured visually on all aggregates observed in the head region of the worms. The experiments were performed by an experimenter blinded to the quantification grouping of worms.

### Locomotion assay

The locomotion speed of worms was analyzed using a multi-worm tracker (MWT), as previously described [24, 60]. Briefly, age-synchronized adult day 2 worms (*n* = 50 for each group) were washed three times in NG buffer and transferred from the NGM culture plate onto the assay plate. The assay plate was a 13 × 10-cm plate filled with agar, which was divided into equal four regions. Regions were surrounded with glycerol, an aversive stimulus for *C. elegans*, to prevent the animals from moving to other regions. The locomotion of the worms was captured using the MWT system, and the images were binarized to calculate the locomotion speed of individual worms.

### Analysis of the MWT data

Analysis of the recordings was performed using Choreography (part of the MWT software) and custom-written scripts to organize and summarize the data. Animal tracks were collected as previously described [24]. Using the MWT software, we drew the trajectory of the worm's movement and measured the average speed of the locomotion.

### Establishment of HeLa-αSyn-mRFP stable line

To generate a stable HeLa-αSyn-mRFP line (gift from Professor T. Yoshimori, Osaka University), HeLa cells were first infected with recombinant viruses prepared from pMRX-ib-αSyn-mRFP using polybrene (TR-1003, Sigma-Aldrich). At 48 h after infection, cells were cultured in selection-medium containing 5 µg/ml blasticidin (A1113902, Gibco). The selection process was conducted for a series of passages by introducing fresh medium and antibiotics to the cells. After 10–14 days, polyclonal populations of blasticidin-selected cells were pooled, expanded, and subjected to fluorescence-activated cell sorting (FACS) using BD FACS AriaIIIu (Becton, Dickinson) to isolate single clones from the top 0.5% RFP-signal cells, followed by manual colony pickup.

### PIP_3_ delivery to Hela αSyn-mRFP cells

Intracellular delivery of PIP_3_ was performed using the PIP_3_ Shuttle PIP™ Kit (P-9039, Echelon Biosciences), with slight modifications to the manufacturer’s protocol. Briefly, HeLa cells stably overexpressing αSyn-mRFP were seeded on a 4-well glass bottom dish (Matsunami, D141400) and incubated overnight at 37 °C. Shuttle PIP carrier 2 (Histone H1) and Bodipy^®^-FL-PIP_3_ were incubated in a 0.2 ml tube in a 1:1 molar ratio for 10 min at room temperature. The complex was diluted with Opti-MEM and added to media covering HeLa cells with a final carrier and PIP_3_ concentration of 5 µM. The following day, the dye-containing media was removed, and cells were washed with phosphate-buffered saline (PBS) before live imaging using SpinSR10 (Olympus) or fixation with 4% paraformaldehyde (PFA) for immunostaining. Confocal images were taken randomly across the entire well at 60× magnification, and the percentage of αSyn puncta-positive cells was quantified. For transient gene overexpression, HeLa cells were seeded and transfected with pcDNA-αSyn-mRFP 24 h prior to delivery of PIP_3_ using Fugene HD transfection reagent (E2311, Promega Corporation), according to the manufacturer’s protocol. For live imaging of lysosomes, cells were incubated with 300 nM LysoTracker™ Blue DND-22 (L7525, Invitrogen) for 1 h and washed three times with PBS before observation.

### Phosphatase inhibitor treatment

SF1670 (B-0350, Echelon Biosciences), dissolved in dimethylsulfoxide (DMSO), was added to the culture medium 24 h after cell seeding. DMSO was used as the vehicle control. After 24 h of treatment, the cells were fixed for immunostaining and imaging.

### Primary neuronal cultures and treatment

Tissue culture plates were coated with poly-l-ornithine (0.2 mg/ml) for 1 h at 37 °C and washed 3 times with autoclaved milli-Q water. C57BL/6 mouse E15.5 pup brains were collected for primary neuronal cultures. Neurobasal media supplemented with B-27, glutamax, penicillin and streptomycin was used for cultures. Glial inhibitor, 5-fluoro-2-deoxyuridine, was added at 3 days in vitro (DIV). A mixture of shuttle PIP carrier 3 and Bodipy^®^-FL-PIP3 was added to the primary neuronal cultures at DIV10. Cells were fixed with 4% paraformaldehyde 4 days after Bodipy^®^-FL-PIP3 treatment, followed by immunostaining of anti-phosphorylated S129 α-syn (1:1000) (EP1536Y, ab51253, abcam), anti-NeuN antibody (1:500) (A60, MAB377, EMD Millipore) and anti-microtubule-associated protein 2 (MAP2) (1:5000) (NB300-213, Novus Biologicals) before microscopic observation using IN Cell Analyzer 6000 (GE healthcare, Chicago, IL, USA). Sixty-four images covering the entire area of culture well were taken at 20× magnification. Automated quantification of the intensity, area and count of phosphorylated α-syn, total area of MAP2 and number of NeuN-positive cells was performed using the software IN Cell Developer Toolbox. For inhibitor experiment, SF1670 (0.5 µM) was added to the primary neuronal cultures at DIV10, and cell fixation was performed at DIV14 followed by immunostaining with purified anti-PtdIns (3,4,5) P3 IgG (1:200) (Z-P345B, Echelon Biosciences), anti-phosphorylated S129 α-syn (1:1000) (EP1536Y, ab51253, abcam) and anti-MAP2 (1:5000) (NB300-213, Novus Biologicals). Confocal images were acquired using SpinSR10 microscope (Olympus, Tokyo, Japan). Following image acquisition, fluorescence intensity of PIP_3_ and pSyn per unit of total MAP2 area were analyzed using ImageJ software. For immunofluorescence assessment of the synaptic localization of PIP_3_ and pSyn, primary antibodies anti-PtdIns (3,4,5) P3 IgG (1:200) (Z-P345B, Echelon Biosciences), anti-phosphorylated α-syn (pSyn#64) (1:500) (015-25191, Wako), anti-SNAP25 (1:250) (1113839, GeneTex) and PSD95 (D27E11) antibodies (1:100) (3450, Cell Signaling Technology) were used.

### Protein purification

Human wild-type (WT) αSyn was purified from *E. coli* as described previously [26, 62]. Briefly, a plasmid containing WT human αSyn was expressed in *E. coli* BL21 (DE3) (69450, Novagen, Merck, San Diego, CA, USA). *E. coli* were suspended in buffer, crushed by sonication, and centrifuged at 8000 rotations per minute (rpm). Streptomycin sulfate (06339-52, Nacalai Tesque, Kyoto, Japan) (final 2.5% [w/w]) was added to the supernatant and centrifuged at 8000 rpm. The supernatant was then heated at 90 °C in a water bath and centrifuged at 20,000 rpm. The supernatant was then (1) precipitated with solid ammonium sulfate (02620-75, Nacalai Tesque, Kyoto, Japan) to 70% saturation, (2) centrifuged at 20,000 rpm, (3) dialyzed overnight, (4) applied onto a Resource-Q column (GE Healthcare, Little Chalfont, UK) with 50 mM Tris–HCl buffer (pH 7.5) containing 0.1 mM dithiothreitol (14112-52, Nacalai Tesque, Kyoto, Japan) and 0.1 mM phenylmethylsulfonyl fluoride (022-15371, FujiFilm Wako Pure Chemical Corporation, Osaka, Japan) as the running buffer, and (5) eluted with a linear gradient of 0–1 M NaCl. αSyn-enriched fractions were pooled and further purified by size exclusion chromatography using a HiLoad Superdex 200 26/600 pg column (GE Healthcare) equilibrated with 50 mM Tris–HCl (pH 7.5) and 150 mM NaCl. The purified fractions were combined and dialyzed against deionized water at 4 °C overnight. Sample solutions were flash-frozen in liquid nitrogen, lyophilized, and stored at − 80 °C until use. The fractions containing αSyn (as determined by SDS-PAGE/Coomasie blue staining) were joint, dialyzed versus deionized water, acidified with 5 mM HCl and loaded onto a Reverse Phase Cosmosil Protein R × 250 mm Preparative Column (Nacalai-Tesque, Kyoto, Japan) and eluted with a linear gradient of 30–90% acetonitrile. The pure fractions were combined and flash-frozen in liquid nitrogen, lyophilized and stored at − 80 ºC until use.

### Lipid binding assay

To assess the direct binding of αSyn with various lipids, a protein–lipid overlay assay was performed using membrane lipid strips (P-6002), PIP strips (P-6001), and Sphingo strips (S-6000) purchased from Echelon Biosciences (Salt Lake City, USA). First, lipid membranes were blocked with chemical-blocking buffer (EzBlock Chemi, AE-1475, ATTO, Tokyo, Japan) for 30 min, and incubated with 0.5 µg/ml of αSyn protein in blocking buffer for 1 h at room temperature with gentle agitation. The strips were washed with TBS-T three times for 10 min with shaking, and then incubated with αSyn antibody (Syn211) (32-8100, Invitrogen) or α/β-synuclein (F-11) antibody (sc-514908, Santa Cruz Biotechnology) for 1 h. After three washes, the strips were treated with anti-mouse IgG HRP-conjugated antibody for 1 h and washed three times. Finally, αSyn binding to each lipid was evaluated by chemiluminescence detection with ECL prime (RPN2232, Cytiva, Tokyo, Japan).

### SUV preparation

SUVs were prepared according to the method described by Suzuki et al. [58]. POPC (L-1618), PI (P-0016), PI-3-P (P-3016), PI-4-P (P-4016), PI-5-P (P-5016), PI-3,4-P_2_ (P-3416), PI-3,5-P_2_ (P-3516), PI-4,5-P_2_ (P-4516), and PI-3,4,5-P_3_ (P-3916) were purchased from Echelon Biosciences. Briefly, lipid mixtures of POPC with respective PIPs (90:10 molar ratio, total concentration at 10 mM) in chloroform were dried under an atmosphere of N2 and lyophilized overnight to remove any trace of organic solvent. The thin lipid film obtained was hydrated in fibrillation buffer (50 mM Tris–HCl pH 7.4, 150 mM NaCl) and sonicated using Bioruptor II (BM Equipment, Japan), with 5 cycles of 30 s of sonication and 30 s of quiescence. The size and integrity of the vesicles were verified using TEM and dynamic light scattering.

### Kinetics of fibril formation followed by thioflavin-T (ThT)

Amyloid fibrils were formed by dissolving lyophilized αSyn in fibrillation buffer, filtering through 0.22 µm membrane, and adjusting to 0.5 mg/mL supplemented with 10 µL ThT (202-01002, FujiFilm Wako Pure Chemical Corporation, Osaka, Japan); all experiments were performed in the presence or absence of 1 mM of POPC-PIPs SUVs. Each reaction mixture (100 µL) was transferred to a 96-well sealed plate (Costar Assay Plate, Corning, USA), with each well containing 35 mg ZrO_2_ beads (YTZ-0.5, Nikkato Corporation) to facilitate the fibril formation. The microplate with the reaction mixtures was subjected to cyclic agitation with a 3 min orbital shaking period at 2000 rpm, followed by a 12 min quiescent period at 37 °C. The kinetics of fibril formation was monitored according to ThT intensity fluorescence (excitation at 450 nm and emission at 485 nm) every 15 min in an MTP-900 microplate reader (Corona Electric Co., Tokyo, Japan). All the reaction conditions were evaluated with at least 15 replicates, and the kinetics was characterized according to the lag time (i.e., the time required to reach a fluorescence value of 500 A.U.) and the maximum ThT intensity (i.e., the highest intensity value in the measuring period).

### Brain lysate preparation

Amygdala sections from the frozen side and amygdala sections from the formalin-fixed side were prepared. Amygdala slices (100 mg) from the brains of patients with PD and MSA were placed into Precellys Lysing tubes (P000912-LYSK 0-A, M&S Instruments, Osaka, Japan), resuspended in 1 mL of fibrillation buffer, and subjected to two cycles of high-speed shaking for 20 s in a lysis and homogenization system (Bertin Instruments, France). Then, the homogenates were transferred into Eppendorf tubes and centrifuged at 2000×*g* for 2 min at room temperature. The concentration of total protein in the supernatant fractions was quantified using the MicroBCA Protein Assay Reagent Kit (23235, Thermo Pierce), and the homogenates were aliquoted and stored at − 80 °C until use.

### Amplification of ɑSyn aggregates from amygdala brain homogenates

A volume of amygdala brain homogenate was added to solutions of ɑSyn to reach a final concentration of 20 µg/mL total protein. Then, 200 µL of 0.5 mg/mL monomeric ɑSyn in the fibrillation buffer containing the amygdala lysate was added into a 96-multiplate (675096, Greiner Bio-One). This was subjected to ultrasonication to accelerate the amyloid formation at an optimized frequency of 30 kHz in cycles of 300 ms of irradiation and 800 ms of quiescence using a Handai Amyloid Burst Inducer (HANABI) equipment (CORONA ELECTRIC, Ibaraki, Japan). ThT fluorescence intensity was recorded as a function of time.

### Transmission electron microscopy

Fibrils were adsorbed onto 400-mesh grids (Nisshin EM Co., Ltd., Tokyo) and negatively stained with 1% phosphotungstic acid (27807-62, Nacalai Tesque, Kyoto, Japan), and their structures were observed using an H-7650 TEM (Hitachi High Technologies Corporation, Tokyo, Japan) operated at 80 kV.

### Proteinase K resistance assay

ɑSyn fibrils (0.5 mg/mL) in the fibrillation buffer were digested using proteinase K (03115887001, Sigma) (1 µg/mL) at 37 °C and agitation at 400 rpm for different time intervals. To stop the reaction, the samples were incubated at 95 °C for 5 min, mixed with loading buffer (1610747, Bio-Rad) (50 mM Tris–HCl, pH 6.8, 4% SDS, 2% β-mercaptoethanol, 12% glycerol, and 0.01% bromophenol blue) and incubated at 95 °C for an additional 10 min. The digestion patterns were analyzed using SDS-polyacrylamide gel electrophoresis, followed by Coomassie Brilliant Blue (11642-31, Nacalai Tesque, Kyoto, Japan) staining. The first five digestion products, B1–B5, were used for analysis. The proteinase K resistance (PKR) score was established as the band intensity ratio between bands B2 and B1 (B2/B1).

### ^1^H-^15^N heterogeneous single-quantum coherence NMR spectroscopy

^1^H-^15^N heterogenous single-quantum coherence (HSQC) NMR measurements were performed using 100 µM ^15^N-labeled ɑSyn dissolved in fibrillation buffer prepared in H_2_O/D_2_O (9:1, v/v). The ^15^N-labeled ɑSyn was expressed in M9 minimal medium containing ^15^NH_4_Cl and purified as described for the unlabeled protein. NMR spectra were acquired at 37 °C on a Bruker AVANCE III HD 600 MHz NMR spectrometer equipped with a 5 mm quadruple resonance cryogenic probe (Bruker Biospin). The data size and spectral width were 256 (t1) × 2048 (t2) and 1338 Hz (ω1, ^15^N) × 9,615 Hz (ω2, ^1^H), respectively. The carrier frequencies of ^1^H and ^15^N were 4.7 and 118 ppm, respectively. The number of scans/FID was 32. The repetition time was 1 s. The peak assignment at pH 7.4 was achieved employing the assignment data reported by El Turk et al. [61]. The chemical shift perturbation (CSP) is calculated as follows:

$$\Delta \delta =\sqrt{{\Delta \delta }_{\mathrm{H}}^{2}+{\left(\frac{1}{8}{\Delta \delta }_{\mathrm{N}}\right)}^{2}},$$

where Δ*δ*_H_ and Δ*δ*_N_ are the chemical shift changes (in ppm) with respect to the H and N axes, respectively. All NMR spectra were processed with Topspin (Bruker Biospin), NMRPipe [15] and NMRFAM-sparky [30].

### Tissue preparation and chromogenic immunohistochemistry (IHC) staining

Clinical profiles of human autopsy cases (disease control patients, *n* = 3; PD patients, *n* = 3) used for chromogenic IHC are shown in Table 1. The midbrains, including the substantia nigra, from the patients were fixed overnight in 4% PFA and then immersed in PBS containing 30% sucrose until sinking. The brain samples were cut into 40-μm-thick sections using a cryostat (CM1850; Leica Microsystems). Free-floating sections were washed in TBS and immersed in a solution of 3% H_2_O_2_ to quench endogenous peroxidase activity. Then, they were incubated with the primary antibody against PtdIns(3,4,5)P3 (1:100) (Z-P345B, Echelon Biosciences) in TBS containing 10% blockace (UKB80, KAC Co., Ltd.) overnight at 4 °C with continuous shaking. The sections were then washed three times in TBS-T and incubated with biotinylated anti-mouse secondary antibody (BA-9200, Vector Laboratories) in TBS-T for 2 h at room temperature. The sections were then incubated with avidin–biotin peroxidase complex (PK-6100, Vector Laboratories) for 1 h. Thereafter, the reaction products were visualized with 3,3-diaminobenzidine tetrahydrochloride. All sections were then washed in TBS, mounted on amino propyltriethoxysilan-coated slides, dried, stained with crystal violet, dehydrated in a graded series of ethanol, cleared in xylene, and coverslipped. Images were obtained using an Eclipse Ni-E microscope (Nikon, Tokyo, Japan).

**Table 1:** Clinical information on the postmortem human brain samples

Case	Age	Sex	PMD (h)	Clinical diagnosis	Cause of death	Lewy pathology [35]	IHC	IF	Lipidomic analysis
Control									
#1	69	M	24	Multiple cerebral infarction, aspiration pneumonia	Respiratory failure	None			〇
#2	67	M	58	Intracerebral hemorrhage, subarachnoid hemorrhage	Cerebral herniation	None			〇
#3	74	M	4	Intracerebral hemorrhage, pneumonia	Respiratory failure	None	〇		〇
#4	84	M	11	Multiple cerebral infarction, Alzheimer's disease, pulmonary aspergillosis	Septic shock	None	〇		〇
#5	83	M	19	Intracerebral hemorrhage, aspiration pneumonia	Respiratory failure	None	〇	〇	〇
#6	88	M	15	Metabolic encephalopathy	Multiple organ failure	None		〇	
#7	65	M	2.5	Chronic liver failure, chronic renal failure	Multiple organ failure	None		〇	
#8	84	F	21	Intracerebral hemorrhage, aspiration pneumonia	Respiratory failure	None		〇	
PD									
#1	83	M	10	PD, pneumonia	Respiratory failure	Limbic			〇
#2	66	M	17	PD, aspiration pneumonia	Respiratory failure	Limbic	〇		〇
#3	86	F	9.5	PD	Suffocation	Limbic	〇		〇
#4	88	M	3	PD, aspiration pneumonia	Respiratory failure	Limbic	〇	〇	
#5	85	M	8	PD, pneumonia	Respiratory failure	Limbic		〇	
#6	84	F	3	PD	Chronic heart failure	Limbic		〇	
#7	79	F	19.5	PD, acute myelocytic leukemia, fungal pneumonia	Multiple organ failure	Brainstem		〇	

### Tissue preparation and immunofluorescence (IF) staining

Clinical profiles of human autopsy cases (disease control patients, *n* = 4; PD patients, *n* = 4) used for IF staining are shown in Table 1. For IF analyses, sections of the midbrains were cut at 40-μm thickness using cryostat (CM1850; Leica Microsystems) and placed onto glass slide. Sections were stored in a sealed slide box at − 80 °C until use. Before immunostaining, the tissue sections were dried at room temperature for 20 min and immersed in pre-cooled acetone (− 20 °C) for 5 min. Fixative was poured off and the tissue sections was dried for 30 min to allow acetone to evaporate from the tissue sections. The slides were rinsed in TBS for 3 changes, 5 min each. The sections were then soaked with blocking agent 10% normal goat serum (NGS) (S-1000 Vector Laboratories) and incubated with the primary antibodies against PtdIns(3,4,5)P3 (1:200) (Z-P345B, Echelon Biosciences), phosphorylated S129 α-syn (1:1000) (EP1536Y, ab51253, abcam) and MAP2 (1:5000) (NB300-213, Novus Biologicals) in 2% NGS a humidified chamber at 4 ℃ overnight. Sections were washed three times before incubation with secondary antibodies for 1 h at room temperature. Alexa Fluor^®^ 405 goat anti-mouse IgG (H + L) antibody (A31553, Thermo Fisher Scientific), Alexa Fluor^®^ 488 donkey anti-mouse IgG (H + L) antibody (A21202, Thermo Fisher Scientific), Alexa Fluor^®^ 594 donkey anti-rabbit IgG (H + L) antibody (A21207, Thermo Fisher Scientific) and Alexa Fluor^®^ Plus 647 goat anti-chicken (A32933, Thermo Fisher Scientific) were used as secondary antibodies. Slides were washed 3 times, 5 min each, in TBS before applying mounting media and coverslip. Confocal mages were obtained using SpinSR10 microscope (Olympus, Tokyo, Japan). Following image acquisition, fluorescence intensity of protein of interest per unit of total MAP2 area were analyzed using ImageJ software.

### Sample preparation for lipidomic analysis

Clinical profiles of human autopsy cases (disease control patients, *n* = 5; PD patients, *n* = 3) used for lipidomic analysis are shown in Table 1. The medulla oblongata and cerebellar cortex of the patients were frozen in powdered dry ice. Frozen samples from the tegmentum of medulla oblongata including the dorsal nucleus of vagus nerve and cerebellar cortex were used for lipidomic analysis.

### Statistical analysis

For statistical comparisons, all data were checked for normality using D’Agostino and Pearson normality test before parametric and non-parametric tests were performed. For parametric test, data were analyzed by one-way and two-way ANOVA followed by Dunnett’s and Tukey’s multiple post hoc test for comparing more than three samples, and two-tailed Student’s *t* test and multiple *t* test for comparing two samples with 95% confidence. For non-parametric analysis, Kruskal–Wallis test with Dunn’s multiple comparisons was performed. Significance was accepted when *p* < 0.05. *p* values are presented as *p < 0.05, **p < 0.01, ***p < 0.001, ****p < 0.0001. All statistical tests were performed using GraphPad Prism 7 (GraphPad Software, Inc, San Diego, CA, USA).

### Ethics statement

This study was approved by the Ethics Committee of Osaka University Hospital (no. 12148) and was conducted in accordance with the Declaration of Helsinki and the Ethical Guidelines for Medical and Health Research Involving Human Subjects endorsed by the Japanese government. All subjects provided informed consent.

## Results

### Depletion of SYNJ1 prompts αSyn accumulation in cultured cells

**Fig. 1:**
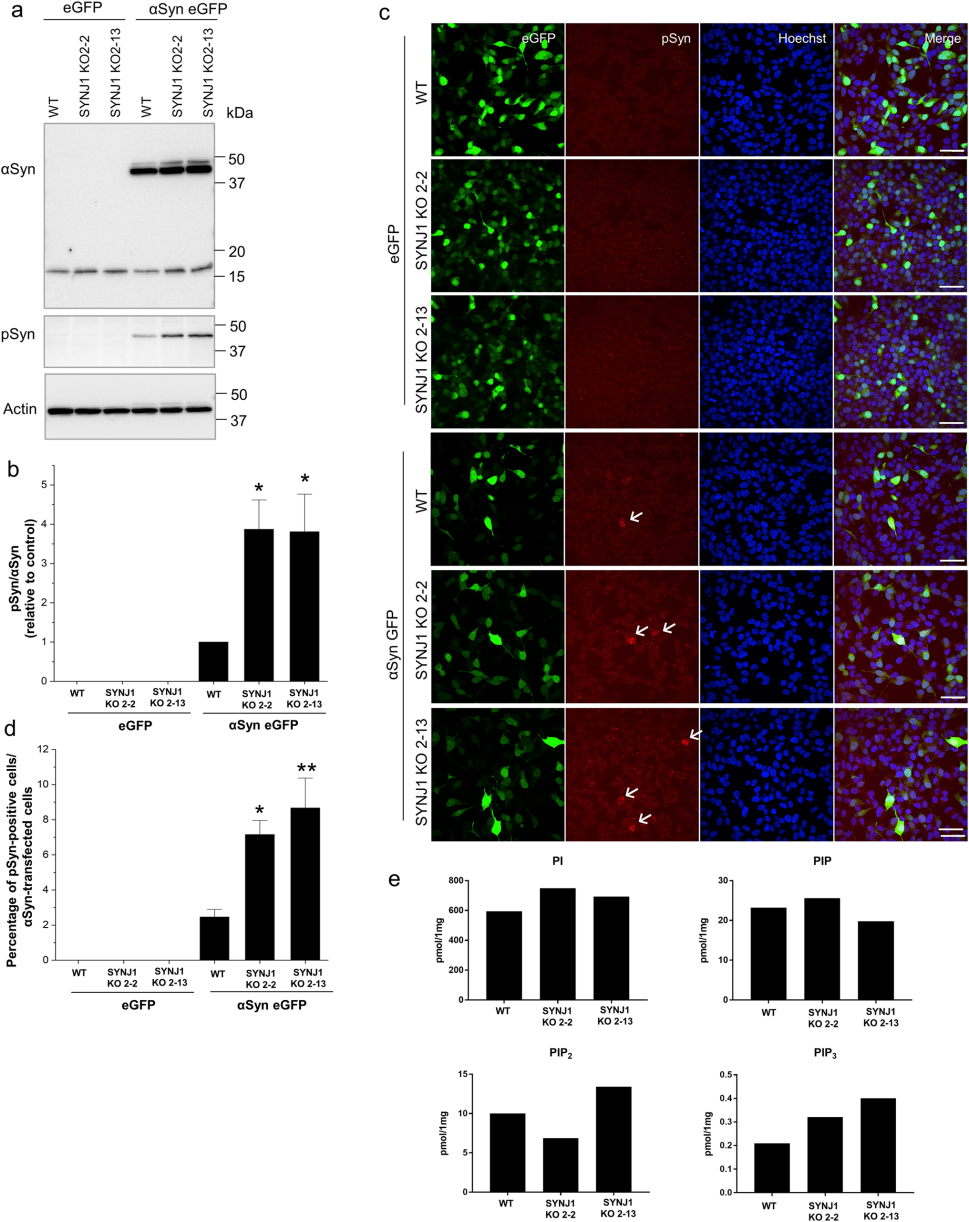
Depletion of SYNJ1 exacerbates pSyn accumulation in αSyn-overexpressing cells. **a** Representative immunoblot showing αSyn, pSyn, and actin levels in SH-SY5Y WT and SYNJ1 knockout cells transiently transfected with eGFP or αSyn-eGFP. **b** The relative pSyn/αSyn band intensity ratio. Data are expressed as the mean ± SEM (*n* = 3); one-way ANOVA followed by Tukey’s post hoc test compared to SH-SY5Y WT-αSyn-eGFP; *p < 0.05. **c** Immunofluorescence staining of pSyn (red) in SH-SY5Y WT and SYNJ1 knockout cells transiently transfected with eGFP or αSyn-eGFP (green). Arrows indicate the pSyn-positive cells. Scale bar = 20 μm. **d** Quantification of percentage of pSyn-positive cells. Data are presented as the mean ± SEM (*n* = 3); one-way ANOVA followed by Tukey’s post hoc test compared to SH-SY5Y WT-αSyn-eGFP; *p < 0.05, **p < 0.01. **e** Lipidomic analysis of total PIPs level in SH-SY5Y WT and SYNJ1 knockout cells

To study how SYNJ1 depletion affects the dynamics of αSyn aggregation, *SYNJ1* knockout (KO) SH-SY5Y cell lines were generated using the CRISPR–Cas9 system. Depletion of endogenous SYNJ1 in *SYNJ1* KO clones (clone 2–2 and 2–13) was confirmed by markedly diminished SYNJ1 expression on immunostaining and loss of the 145 kDa isoform band in the KO clones on western blotting (Supplementary Fig. 1a, b, online resource). The cells were then transfected with either an eGFP-expressing control plasmid or an αSyn-eGFP-expressing plasmid. Overexpression of αSyn caused accumulation of Ser129-phosphorylated αSyn (pSyn), which was exacerbated by the loss of SYNJ1, as revealed by immunoblotting (Fig. 1a, b) and immunofluorescence analysis (Fig. 1c, d). In line with the functional role of SYNJ1 as a polyphosphoinositide phosphatase, lipidomic analysis of PIPs revealed the level of PIP_3_ but not the other PIPs was higher after genetic ablation than in the control (Fig. 1e). Immunostaining for PIP_3_ also showed more robust and intense intracytoplasmic staining in the *SYNJ1* KO clones than the control (Supplementary Fig. 1c, d, online resource). These results demonstrate a correlation between the loss of SYNJ1 function, which leads to the accumulation of PIP_3_, and αSyn aggregation*.*

### Mutation in *SYNJ1* increases αSyn aggregation and induces locomotory defects in *C. elegans*

**Fig. 2:**
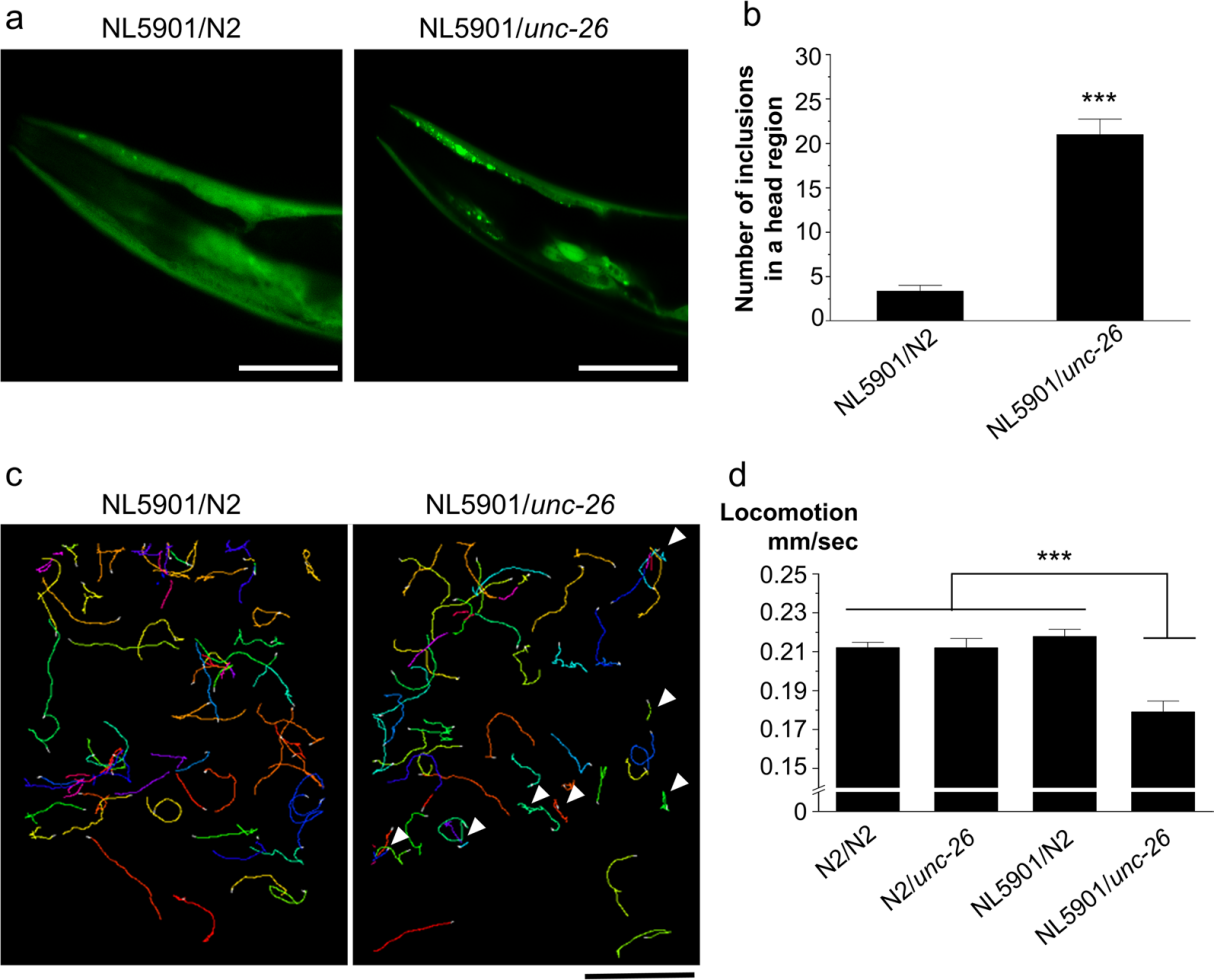
SYNJ1 haploinsufficiency causes αSyn accumulation and locomotor defect in *C. elegans*. **a** Representative images of the heads of αSyn-expressing model of *C. elegans*, crossed with (left) wild type or (right) SYNJ1 (unc-26) mutant. Scale bar = 50 μm. **b** Quantification of the number of obvious puncta in the head region of the models. Data are expressed as the mean ± SEM (*n* = 14 per group); Student’s *t* test; ***p < 0.0001. **c** A representative trajectory of NL5901/N2 (left) and NL5901/*unc-26* (right) worms for 100 s. A 13 × 10 cm agar-filled plate was used for the assay, and images were captured using Toshiba-Teli Ultra-High-resolution 12 M pixel CMOS sensor camera-link camera. Arrowheads represent immobile worms. Scale bar = 2 cm. **d** Comparison of the locomotion speed of the worms. Data are expressed as the mean ± SEM (*n* = 50 per group); two-way ANOVA with Dunnett’s post hoc analysis compared to controls; ***p < 0.001

To assess the effect of SYNJ1 dysfunction on αSyn aggregation in vivo, we employed *C. elegans* model. First, we crossed NL5901, a transgenic strain expressing wild-type “human” αSyn protein in body wall muscle [20], to unc-26 (s1710), a strain with SYNJ1/unc-26 loss-of-function by a five-nucleotide deletion that results in a protein truncated within the NH2-terminal Sac1 domain [21]. NL5901 shows age-dependent formation of αSyn aggregation and has been widely used to evaluate the pathophysiology of αSyn [20]. The homozygotes of unc-26 show severe kinker phenotype with a depletion of vesicles at synapses and an accumulation of coated vesicles [21]. The heterozygotes of the NL5901 and unc-26 lines exhibited significantly more abundant αSyn aggregation than did those of the NL5901 crossed with wild-type strain N2 (Fig. 2a, b). Movement tracking of the worms by multi-worm tracker (MWT) showed that the NL5901/unc-26 moved less than NL5901/N2 with some worms being almost immobile (Fig. 2c). Quantification of locomotor speed revealed that heterozygotes of the N2/unc-26 and NL5901/N2 showed no significant difference compared to the wild type. On the other hand, a significant decrease in locomotory speed was observed in NL5901/unc-26 (Fig. 2d), indicating locomotor defect attributed to the toxicity of aggregated αSyn.

### PIP_3_ accumulation in cultured cells promotes intracellular formation of αSyn inclusions

**Fig. 3:**
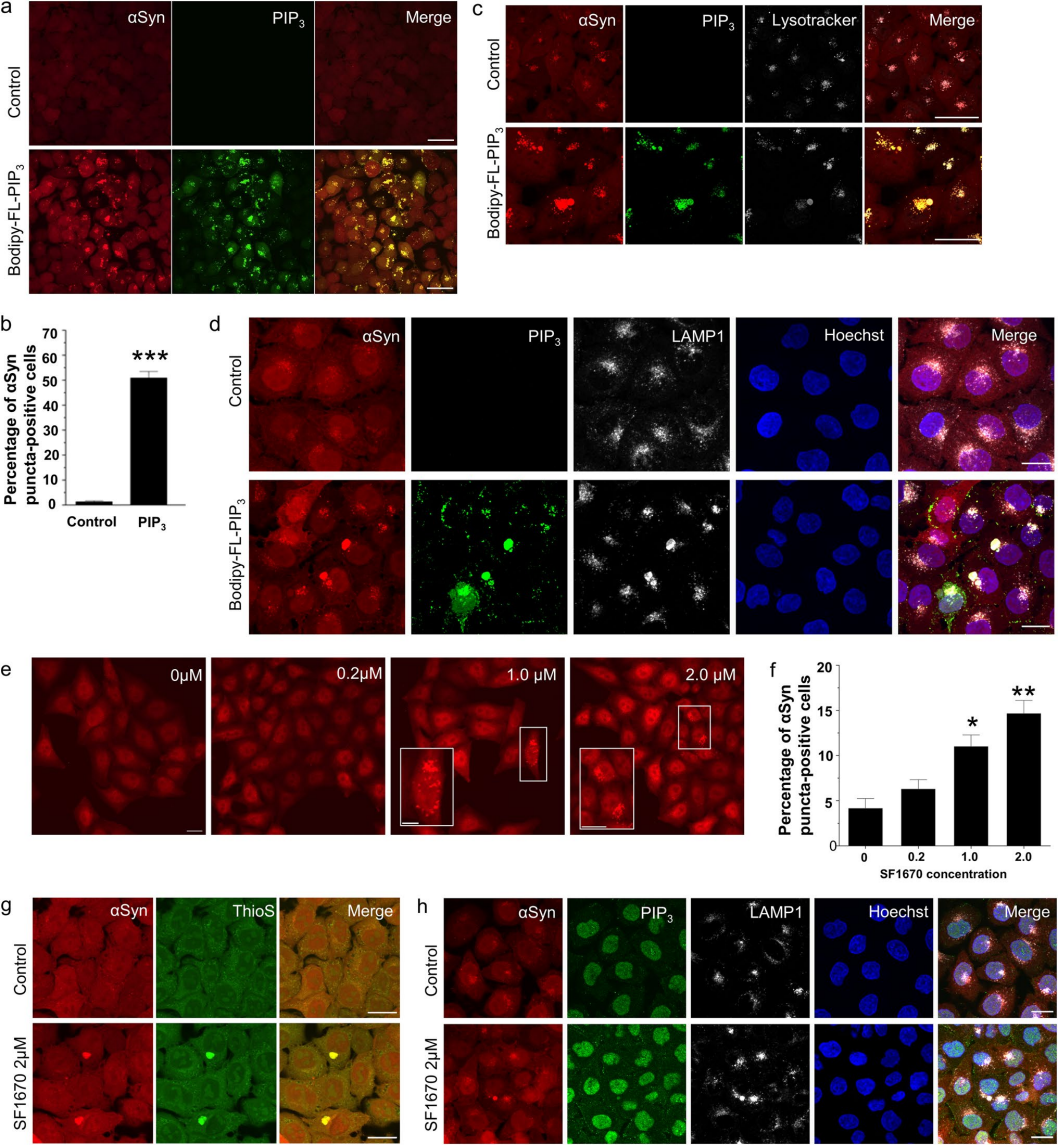
Increasing cellular PIP_3_ level either exogenously or endogenously causes intracellular formation of αSyn inclusions. **a** Representative confocal images of Hela-αSyn mRFP stable cell line (red) treated with BODIPY-FL^®^PIP_3_ (green) using shuttle PIP carrier. Scale bar = 20 μm. **b** Quantification of percentage of αSyn-RFP puncta-positive cells following delivery of Bodipy-FL^®^PIP_3_. Data are presented as the mean ± SEM (*n* = 3); paired Student’s *t* test compared to control; ***p < 0.001. **c** Staining of control and Bodipy-FL^®^PIP_3_-treated Hela-αSyn mRFP cells with LysoTracker (gray) and **d** lysosomal marker LAMP1 (gray). Cells are counterstained for nuclei with Hoechst (blue). Scale bar = 10 μm. **e** Confocal images showing formation of αSyn puncta in HeLa-αSyn-mRFP cells treated with various concentrations of SF1670, an inhibitor of PIP_3_ phosphatase PTEN. Scale bar = 10 µm. Magnified images of the cells with puncta are shown in bottom left (white rectangles, Scale bar = 5 µm). **f** Quantification of percentage of αSyn-RFP puncta-positive cells. Data are presented as the mean ± SEM (*n* = 3); one-way ANOVA followed by Dunnett’s post hoc test compared to control; *p < 0.05, **p < 0.01. **g** Thioflavin S (green) staining of control and SF1670-treated HeLa-αSyn-mRFP cells (red). Scale bar = 10 μm. **h** Double immunofluorescence staining of PIP_3_ (green) and LAMP1 (gray) in control and SF1670-treated HeLa-αSyn-mRFP cells (red). Cells are counterstained for nuclei with Hoechst (blue). Scale bar = 10 μm

Functional loss of SYNJ1 promotes the pathological aggregation of αSyn via the upregulation of its substrate PIP_3_. To determine if PIP_3_ has a direct effect on αSyn aggregation, we treated HeLa cells stably overexpressing αSyn-RFP (hereafter referred to as HeLa-αSyn-RFP) with a water-soluble analog of PIP_3_ labeled with the green fluorophore (Bodipy^®^-FL; hereafter referred to as Bodipy-FL-PIP_3_) using histone as carrier. Intracellularly delivered PIP_3_, which predominantly accumulated in the cytoplasm, induced substantial formation of αSyn-RFP inclusions (Fig. 3a, b). In an assay using transient transfection of αSyn-RFP to HeLa cells, RFP inclusions were not observed in the non-transfected cells, regardless of the accumulation of Bodipy-FL-PIP_3_, thereby excluding the possibility of fluorescent leakage from Bodipy-FL (Supplementary Fig. 2a, online resource). Lysotracker staining demonstrated diffuse or small puncta-like structures in control cells, but structurally enlarged lysosomes colocalized with αSyn-RFP inclusions in PIP_3_-treated cells (Fig. 3c). Similarly, PIP_3_-induced αSyn inclusions showed immunoreactivity for the lysosomal markers, LAMP1 and LAMP2, suggesting the sequestration of αSyn aggregates into lysosomes (Fig. 3d and Supplementary Fig. 2b, online resource).

We further examined if the upregulation of endogenous PIP_3_ levels by a phosphatase inhibitor induces αSyn aggregation. HeLa-αSyn-RFP were treated with SF1670, a phosphatase and tensin homolog deleted from chromosome 10 inhibitor, to inhibit phosphatase activity that converts PIP_3_ to PI-4,5-P_2_, thus elevating intracellular PIP_3_ levels. Immunofluorescence staining confirmed SF1670-induced upregulation of PIP_3_ (Supplementary Fig. 3a, b, online resource). SF1670 treatment led to the formation of αSyn-RFP inclusions in a concentration-dependent manner (Fig. 3e, f). Thioflavin-S-positive staining indicates that these inclusions contain fibrillar material with a β-pleated sheet conformation (Fig. 3g). Double immunofluorescence staining revealed that PIP_3_-induced αSyn-RFP inclusion occasionally colocalized with the lysosomal markers, LAMP1 and LAMP2 (Fig. 3h and Supplementary Fig. 3c, online resource). These data indicate that increasing cellular PIP_3_ levels either exogenously or endogenously causes pathological accumulation of αSyn, which is incorporated into lysosomes.

### PIP_3_ hastens the formation of aggregates from endogenously expressed αSyn in primary neurons

**Fig. 4:**
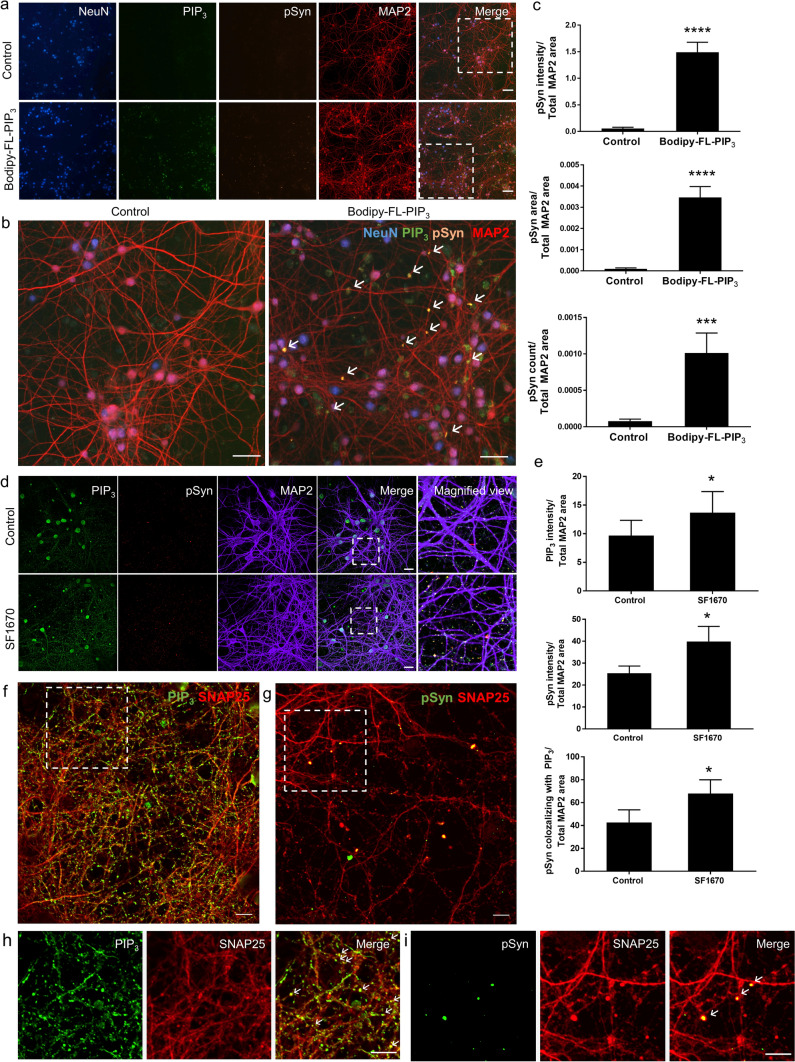
Accumulation of pSyn in primary mouse cortical neuronal culture following increase of cellular PIP_3_ level. **a** Representative IN Cell Analyzer images of primary mouse cortical neurons treated with BODIPY-FL^®^PIP_3_ (green) and subjected to triple immunofluorescence staining for NeuN (blue), pSyn (orange) and MAP2 (red). Scale bar = 100 μm. **b** Magnified view of the areas marked with white dashed-line squares. Scale bar = 25 μm. **c** Quantification of pSyn signal intensity, area of staining and count per unit area of MAP2 in control and Bodipy-FL^®^PIP_3_-treated cells. Data are presented as the mean ± SEM (*n* = 6); paired Student’s *t* test compared to control; ***p < 0.001, ****p < 0.0001. **d** Representative confocal images of primary mouse cortical neurons with control and SF1670 treatment. Cells were subjected to triple immunofluorescence staining for PIP_3_ (green), pSyn (red) and MAP2 (indigo). Scale bar = 20 μm. Magnified views of dashed boxed areas are shown in the right panel. **e** Quantification of signal intensity of PIP_3_, pSyn and pSyn colocalizing with PIP_3_ per unit area of MAP2 of control and SF1670-treated cells. Data are presented as the mean ± SEM (*n* = 3); paired Student’s *t* test compared to control; *p < 0.05. **f**–**i** Double immunofluorescence staining of primary mouse cortical neurons for **f** PIP_3_ (green) and SNAP25 (red) and **g** pSyn (green) and SNAP25 (red). Magnified view of dashed boxed areas are shown in **h**, **i**. Scale bar = 10 μm

To strengthen our finding, we examined the effect of PIP_3_ on αSyn aggregation in primary neuronal culture, a more reliable and ideal system as the cultured cells retain morphological and neurochemical properties that are comparable to those of neurons in the brain. Briefly, primary mouse cortical neurons were prepared from E15.5 C57BL/6 wild-type embryos and these primary neurons expressing endogenous levels of αSyn were treated with Bodipy-FL-PIP_3_ at DIV10 and incubated until DIV14. By immunocytochemistry, we detected enhanced accumulation of pSyn in the neuronal processes upon treatment with Bodipy-FL-PIP_3_ (Fig. 4a, b), as shown by the increase of pSyn intensity, area and count per unit area of MAP2 (Fig. 4c).

We also assessed the effect of PTEN inhibitor SF1670 treatment on PIP3 upregulation and endogenous αSyn aggregation in primary mouse cortical neurons. We observed increased neuronal PIP_3_ level, higher abundance of pSyn and PIP_3_-induced pSyn inclusions in SF1670-treated neurons compared to control, even though the changes were not as prominent as in the case of Bodipy-FL-PIP_3_ treatment (Fig. 4d, e). Interestingly, the increased endogenous PIP_3_ by the treatment were abundantly expressed in cell processes observed as a dotted pattern, presumably due to presence in the synaptic boutons or dendritic spines. To further characterize its synaptic localization, we performed immunostaining with the presynaptic and postsynaptic markers, Synaptosomal-associated protein-25 (SNAP25) and postsynaptic density protein 95 (PSD95). PIP_3_ predominantly colocalized with SNAP25 (Fig. 4f, h) but not PSD95 (Supplementary Fig. 4a, online resource) in the neuronal processes, indicating its location in the presynaptic boutons. The majority of the dot-shaped pSyn inclusions were also found to be localized in the presynaptic region (Fig. 4g, i, Supplementary Fig. 4b, online resource). These data obtained from primary neurons demonstrate that the elevated cellular PIP_3_ can recruit endogenous αSyn into forming pathologic inclusions in the presynaptic region in neurons.

### Protein–lipid overlay assay reveals phosphatidylinositol phosphates strongly interact with ɑSyn

**Fig. 5:**
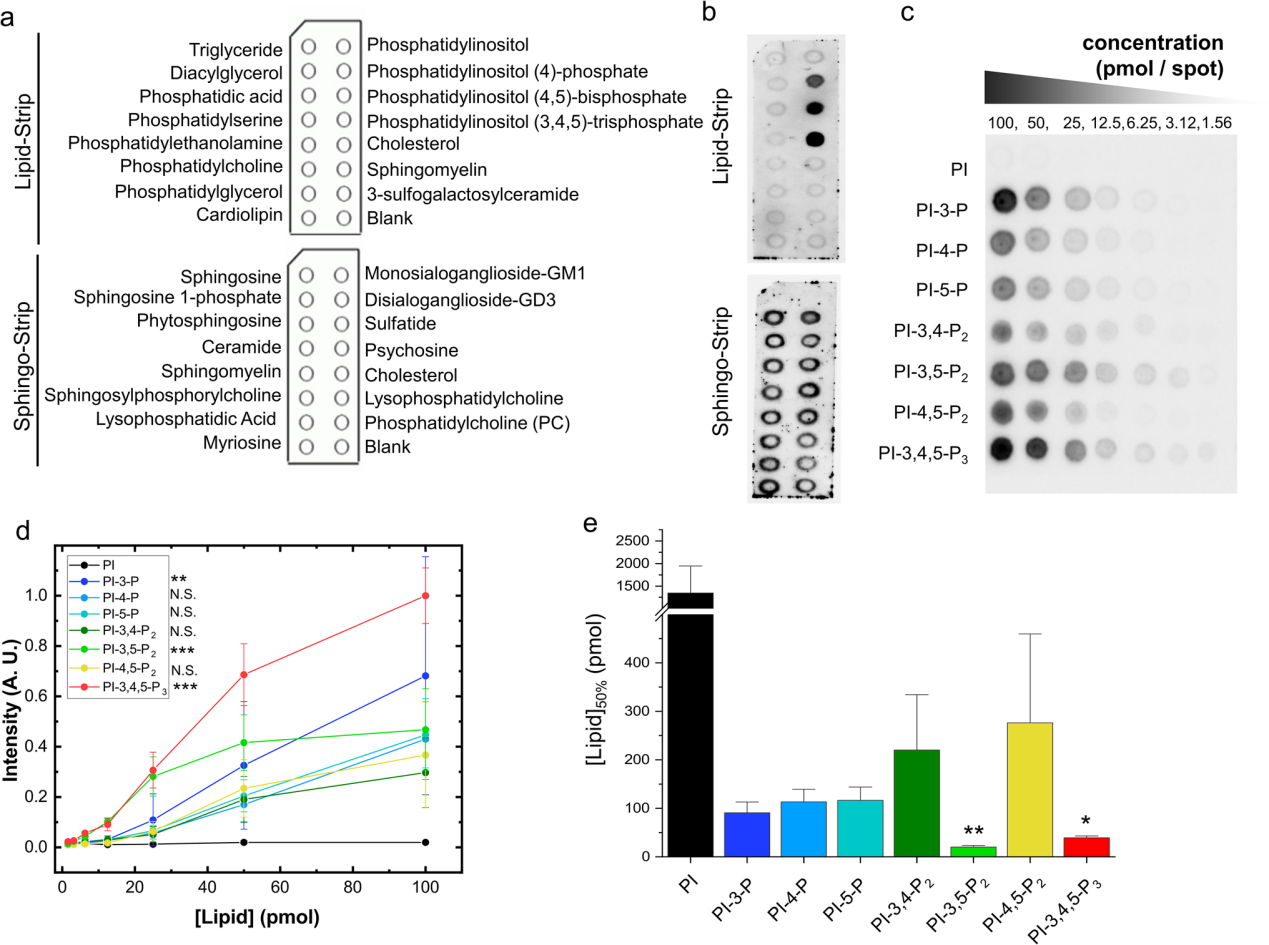
Determination of lipid binding profile of αSyn using lipid overlay assays. **a** Schematic layout of the lipid strip and Sphingo strip, with spots of 100 pmol of different lipids. **b** Representative images of dot-blotting of strip membranes with 0.5 μg/mL αSyn. **c** PIP array displays the concentration-dependent binding of αSyn (0.5μg/mL) to phospholipids. **d** Quantification of the signal intensities of PIP array. Data are shown as the mean ± SEM (*n* = 3); two-way ANOVA followed by Dunnett’s post hoc test compared to PI; **p < 0.01, ***p < 0.001. **e** Determination of the strength of the interaction between αSyn and PIPs employing the binding parameter [Lipid]_50%_. Data are shown as the mean ± SEM of three independent assays (*n* = 3); Kruskal Wallis followed by Dunn’s post hoc test compared to PI; *p < 0.05, **p < 0.01

To understand how PIP_3_ initiates αSyn aggregation, we turned to in-vitro-based assays to investigate if PIP_3_ interacts with αSyn. First, we examined the interactions between several lipids and αSyn using a membrane strip system, which consists of a hydrophobic membrane that has been spotted with 100 pmol of different biologically important lipids found in cell membranes (Fig. 5a). A total of 28 lipids were evaluated, and the results showed that PIPs interacted strongly with αSyn in a dose-dependent manner (Fig. 5b, Supplementary Fig. 5a, b, online resource). The amount of αSyn bound to PIPs could only be detected using an antibody that recognized the C-terminus, but not the N-terminus region of αSyn (Supplementary Fig. 5c, online resource), suggesting that αSyn interacted with PIPs through its N-terminal domain. Additionally, to verify the specificity of the PIPs-αSyn interaction, the amyloidogenic protein beta2-microglobulin was used as a control in the membrane strip system, and the results showed no interaction with the PIPs (Supplementary Fig. 5d, online resource). To further compare the binding affinity of the PIPs for αSyn, we used a PIP array spotted with a concentration gradient of PI and PIPs, each with a different level and position of phosphorylation. Among the PIPs evaluated, PIP_3_ showed the strongest interaction with αSyn, followed by PI-3,5-P_2_ and PI-3-P, as determined by the signal intensity (Fig. 5c, d). Assessment of the binding parameter [Lipid]50%, a value that was obtained by fitting the signal intensities to the sigmoidal Boltzmann equation (Supplementary Fig. 5e, online resource) showed that the [Lipid]50% value was significantly lower than that of PI in PI-3,5-P_2_, and PIP_3_ (Fig. 5e).

### ɑSyn interacts with PIP_3_ through its positively charged N-terminal and hydrophobic NAC regions

**Fig. 6:**
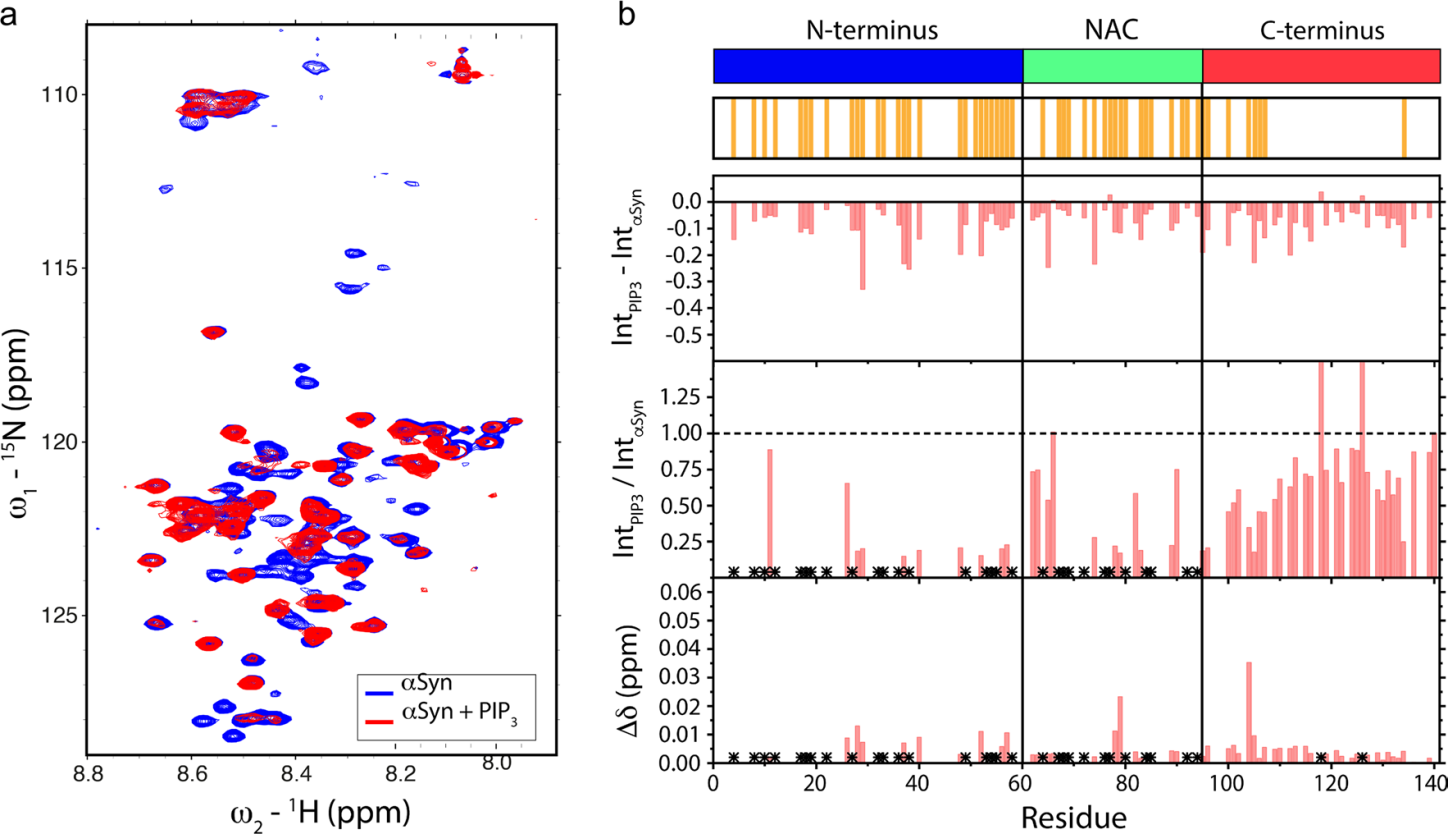
αSyn interaction with PIP_3_ involves the N-terminal domain and the NAC region.** a** ^1^H-^15^N HSQC spectra of αSyn in the presence and absence of monomeric PIP_3_. **b** Analysis of intensity difference, intensity ratio, and chemical shift perturbation between the αSyn N–H nuclei recorded in the presence and absence of monomeric PIP_3_. Top: residues that exhibit the most relevant changes upon interaction with PIP_3_. *Represents signals that disappeared in the presence of PIP_3_

To determine the molecular contacts involved in the interaction between PIP_3_ and αSyn, we performed ^1^H-^15^N HSQC measurement for ^15^N labeled αSyn solutions in the presence and absence of ten equivalents of monomeric PIP_3_ at physiological ionic strength (Fig. 6a). Analysis of the peak intensities normalized using the ^1^H-^15^N HSQC spectrum of αSyn in the absence of PIP_3_, especially the IntPIP_3_/IntαSyn ratio, indicated that the most affected regions were the N-terminal domain and the NAC region, where several peaks disappeared upon the interaction, while most of the C-terminal residue peaks remained detectable (Fig. 6b). The driving forces of the binding seem to involve electrostatic contacts between the negative charges of the three phosphate groups in PIP_3_ and the positively charged N-terminal domain in αSyn. However, the hydrophobic NAC region of αSyn also exhibited important changes.

### PIP_3_ accelerates the fibrillation of ɑSyn and induces the formation of PD-like fibril polymorphisms

**Fig. 7:**
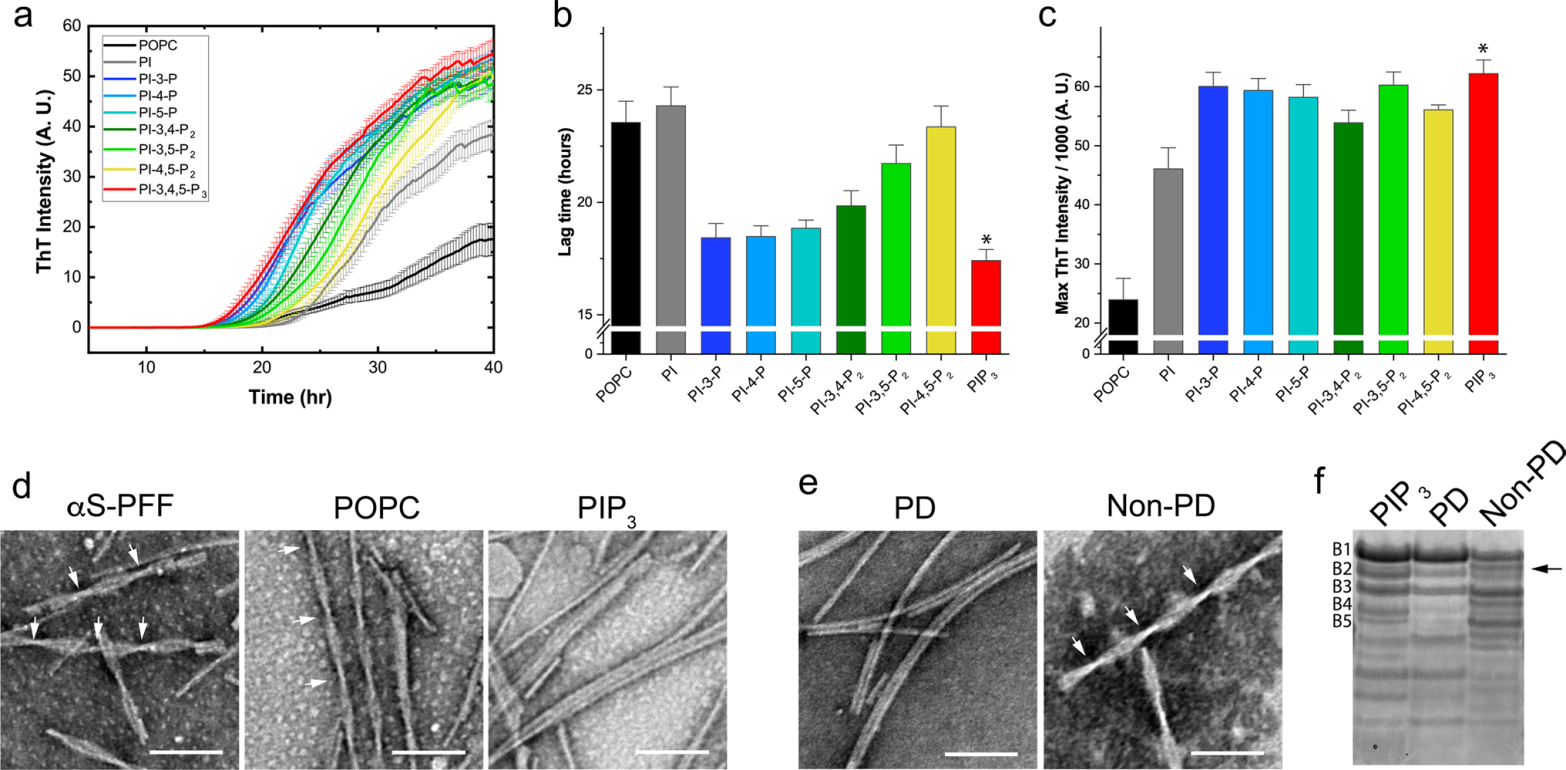
Effect of phosphatidylinositol derivatives on the fibril formation of αSyn. **a** ThT-monitored αSyn fibril formation in the presence of POPC SUVs containing 10% of each phosphatidylinositol derivative. **b** Lag time values of fibril created in the presence of the indicated POPC-phosphatidylinositol-derived vesicles. Bars represent the mean ± SEM (*n* = 3); one-way ANOVA followed by Dunnett’s test compared with POPC; *p < 0.05. **c** Maximum ThT intensity values of fibril created in the presence of the indicated POPC-phosphatidylinositol-derived vesicles. Bars represent the mean ± SEM (*n* = 3); Kruskal–Wallis followed by Dunn’s test compared with POPC; *p < 0.05. **d** TEM visualization of αSyn fibrils obtained in the absence (left) and presence of POPC SUV (middle) and POPC-PIP_3_ SUV (right). Scale bar = 200 nm. Arrowheads represent twist in the fibrils. **e** TEM visualization of fibrils amplified from the brain of PD and non-PD patients. Scale bar = 200 nm. Arrowheads represent twist in the fibrils. **f** PKR digestion patterns of fibrils created in the presence of PIP_3_ vesicles and fibrils amplified from PD and non-PD brains. Bands numbered from B1 to B5 are employed for analysis and comparison

To determine the effect of PIP interactions on the fibrillation of αSyn, we created small unilamellar vesicles (SUVs) composed of 1-palmitoyl-2-oleoyl-glycero-3-phosphocholine (POPC) and PIPs with different levels and positions of phosphorylation. These SUVs were added to the solutions of αSyn in fibrillation buffer, and the fibril formation was monitored by thioflavin-T (ThT) fluorescence intensity (Fig. 7a). We observed that the lipid vesicles containing PIP_3_ (POPC-PIP_3_ SUVs) induced the fastest fibrillation reaction and the highest ThT fluorescence intensity. Meanwhile, SUVs without any PIPs (POPC SUVs) generated fibrils with slower kinetics and the lowest ThT intensity values (Fig. 7b, c). In the case of SUVs with mono- and di-phosphorylated PIPs, the kinetics was faster than that of the POPC SUVs or SUVs containing PI (POPC-PI SUVs), but slightly slower than those of POPC-PIP_3_ SUVs, with high ThT intensity values for all the cases (Fig. 7b, c).

To understand the effect of PIP interactions on fibril polymorphisms, we evaluated the morphological and biochemical characteristics of the fibrils. Transmission electron microscopy (TEM) examination revealed that the fibrils created with POPC SUVs exhibited a dominant twisted morphology, similar to that of the pure αSyn preformed fibrils (PFFs). Meanwhile, the fully phosphorylated PIP_3_-derived fibrils exhibited a rod-like morphology with parallel-protofilaments aligned (Fig. 7d). Fibrils formed in the presence of mono- and di-phosphorylated PIPs showed a twisted morphology with a longer pitch length than that observed in the POPC SUVs (Supplementary Fig. 6a, online resource) or in the absence of lipids.

Recent studies focusing on αSyn polymorphisms and their relationship to specific synucleinopathies have shown that αSyn fibrils amplified from PD brains using the real-time quaking-induced conversion technique (RT-QuIC) showed a rod-like morphology [52]. Remarkably, we found that the fibrils formed in the presence of PIP_3_ were considerably similar to the fibrils amplified from the PD brain, while fibrils amplified from the control brain(s) did not show such rod-like morphology (Fig. 7e). In addition, the proteinase K digestion profiles of the created fibrils were also evaluated to biochemically characterize the different morphologies induced by the PIPs, employing the pattern properties of the first five highest molecular weight bands (bands B1–B5, Supplementary Fig. 6b, online resource). The fibrils with a twisted morphology, including the αSyn control fibrils without lipids (αS-PFF), the fibrils created in the presence of POPC SUVs (POPC), and the fibrils amplified from the brains of non-PD patients showed a split of band B2 into a double-band with low intensity. In addition, band B4 of these twisted fibrils had relatively weaker intensities than did band B5 (Fig. 7f, Supplementary Fig. 6b, online resource). Interestingly, the PIP_3_-derived fibrils and the fibrils amplified from PD brains, which have a rod-like morphology, showed strong intensity for band B2. Band B4 of these rod-like fibrils was more intense than that of B5 (Fig. 7f, Supplementary Fig. 6b, online resource).

Collectively, our in vitro biochemical assays indicate that PIP_3_ interacts with the N-terminal and NAC regions of αSyn, promotes faster aggregation of αSyn, and induces the formation of fibrils with morphological and biochemical characteristics similar to those of PD-derived fibrils.

### Accumulation of PIP_3_ in pathological αSyn inclusions found in the brains of PD patients

**Fig. 8:**
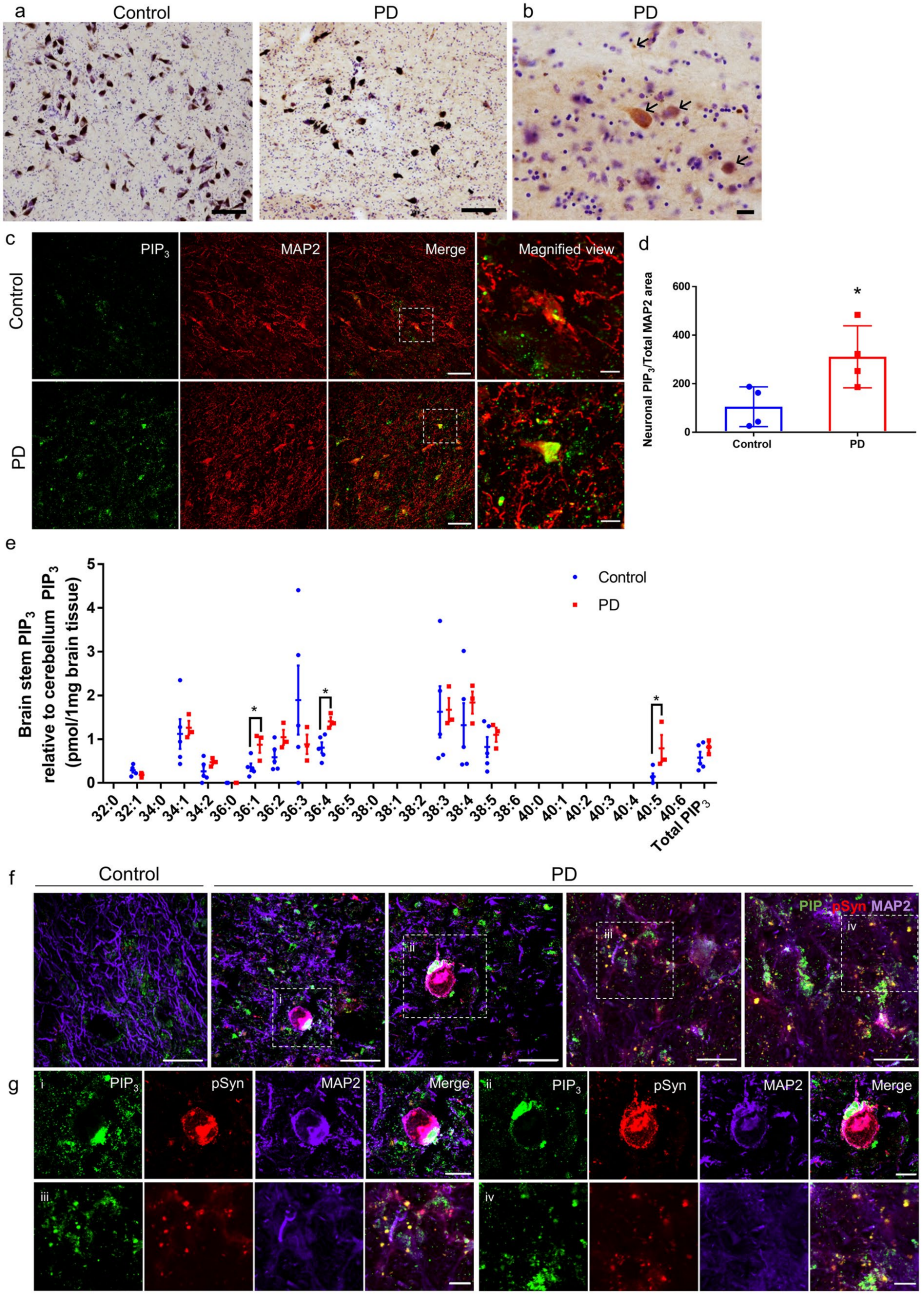
Immunohistochemical and lipidomic analyses of PIP_3_ using postmortem brain tissues. **a**, **b** Representative low magnification (**a**: ×100) and high magnification (**b**: ×400) images of PIP_3_ immunohistochemistry with Nissl staining in the substantia nigra of control and PD patients. Scale bar: **a** = 100 µm, **b** = 25 µm. Arrows show positive PIP_3_ staining. **c** Double immunofluorescence staining of PIP_3_ and MAP2 in the midbrain sections of control and PD patients. Scale bar = 50 µm. The dashed boxed areas are enlarged on the right panel. Scale bar = 20 µm. **d** Quantification of signal intensity of neuronal PIP_3_ per unit area of MAP2 in control and PD groups. Data are presented as the mean ± SEM (*n* for control = 4, *n* for PD = 4); unpaired Student’s *t* test compared to control; *p < 0.05. **e** Lipidomic profile of PIP_3_ species in the postmortem brain stem samples of control and PD patients. PIP_3_ level in the non-affected area cerebellum was used as internal control. Data are presented as the mean ± SEM in an interleaved scatter plot (*n* for control = 5, *n* for PD = 3); multiple *t* test—one per row compared to control; *p < 0.05. **f** Triple immunofluorescence staining of PIP_3_ (green), pSyn (red) and MAP2 (indigo) in the midbrain sections of control and PD patients. Scale bar = 50 µm. The dashed boxed areas are enlarged in **g**. Scale bar = 20 µm

To study the relationship between PIP_3_ and PD pathogenesis, we performed immunohistochemistry staining to evaluate the changes in PIP_3_ levels in postmortem brain samples of age-matched non-neurodegenerative disease control and sporadic PD patients with limbic Lewy pathology (Table 1). Chromogenic-based immunohistochemistry showed more intense PIP_3_ staining in the substantia nigra of PD patients than in control subjects (Fig. 8a, b) and inclusion-like accumulation of PIP_3_ in PD patients (Supplementary Fig. 7, online resource). Quantitative analysis of double immunofluorescence staining of PIP_3_ and neuronal marker MAP2 in the midbrain sections of control and PD patients revealed a higher level of neuronal PIP_3_ immunoreactivity in PD samples compared to controls (Fig. 8c, d).

To obtain a more profound insight into the dysregulation of PIP_3_ in PD, we performed lipidomic analysis comparing the changes of PIP_3_ level in the tegmentum of medulla oblongata, the most inferior part of brain stem where the Lewy pathological changes consistently develop, of control (*n* = 5) and PD (*n* = 3) patients (Table 1). PIP_3_ level in non-affected area cerebellum was used as internal control due to the variations in postmortem delay (PMD) among the samples. As shown in the lipidomic profile (Fig. 8e), we observed merely a tendency of higher total PIP_3_ level in PD group compared to control. However, more comprehensive analysis on the PIP_3_ species categorized based on their acyl chain profile presented in the form of total number of carbon atoms and number of double bonds (carbon atoms: double bonds) showed that rather than the entire lipid class, a few individual PIP_3_ species including 36:1 PIP_3_, 36:4 PIP_3_ and 40:5 PIP_3_ were significantly elevated in PD group compared to control.

To investigate if the sporadic PD cases included in this study belong to the sub-group which has markedly low SYNJ1 levels, we performed DAB immunostaining for SYNJ1 in the control and PD cases (Table 1) but observed no significant difference between the two groups (Supplementary Fig. 8a, online resource). Quantification following double immunofluorescence staining for SYNJ1 and MAP2 of control and PD brain samples (Table 1) also showed no significant difference between the two groups (Supplementary Fig. 8b,c, online resource), indicating that the PD cases used in this study deviate from the abovementioned sub-group. However, our results show that even with no notable changes in SYNJ1 level, PIP_3_ accumulation is a common phenotype observed in PD cases. One explanation could be loss of SYNJ1 represents an upstream contributing factor to PIP_3_ buildup in PD brain samples but is not the only reason.

Importantly, focusing on how PIP_3_ accumulation impacts αSyn aggregation in PD pathogenesis, triple immunofluorescence staining revealed the presence of PIP_3_- and pSyn-immunoreactive inclusions in the cytoplasm of MAP2-positive neurons and dotted inclusions in the interstitial region of the midbrain of PD patients, but almost no pSyn immunoreactivity in the control sections, ruling out the possibility that these structures are artifacts (Fig. 8f, g). The round shaped dotted PIP_3_- and pSyn-immunoreactive structures found in the interstitial region of the brain were reminiscent of the αSyn inclusions observed in primary neuronal cultures (Fig. 8giii, iv). Taken together, these data obtained from postmortem human brains imply that PIP_3_ accumulation might be involved in the process of pathological αSyn inclusions formation and thereby contribute to the pathogenesis of PD.

## Discussion

Genetic and pathological studies have revealed that lipid dysregulation plays a key role in the pathomechanisms of αSyn aggregation in patients with PD [28, 50, 59]. In this study, we demonstrate that functional loss of lipid phosphatase SYNJ1 promotes the pathological aggregation of αSyn via the dysregulation of its substrate PIP_3_. Concomitantly, we identify PIP_3_ as a novel αSyn interactor and aggregation inducer and relate its dysregulation to PD.

Mutations in SYNJ1 have been identified in an early-onset autosomal recessive form of PD (PARK20) [45]. The pathomechanisms of PARK20 have been related to the dysfunction of synaptic vesicle recycling and the autophagy system [12, 16, 40]. However, the direct interplay between SYNJ1 and αSyn aggregation is not fully understood. Our results indicate that SYNJ1 deficiency causes intracellular αSyn aggregation mediated by the accumulation of PIP_3_ and locomotor decline in *C. elegans* models. Indeed, Synj1 haploinsufficiency mice have been reported to exhibit PD-like pathologies comprising αSyn accumulation, impaired autophagy and dopaminergic terminal degeneration as well as age-dependent motor function abnormalities. In the study, the authors showed the elevation of 5′-phosphatase substrate, PI-4,5-P_2_, in the Synj1 haploinsufficiency mice but did not investigate the level of PIP_3_. Importantly, down-regulation of SYNJ1 transcript could be observed in a subset of sporadic PD brains, implicating involvement of SYNJ1 deficiency and the upregulation of its PIP substrates in αSyn accumulation in sporadic PD [39]. Starting off with genetic approach, we demonstrate PIP_3_ is one of the lipid candidates that can initiate αSyn aggregation.

PIPs make up only a small fraction of cellular phospholipid and yet they play important roles in a wide range of cellular processes, including membrane dynamics, trafficking, and intracellular signaling, with each PIP exhibiting distinct subcellular localization and function [6, 14]. For example, PI-4,5-P_2_ and PIP_3_ are found at the plasma membrane, PI-3,4-P_2_ is largely localized at the plasma membrane and in the early endocytic pathway, PI-3-P is concentrated in early endosomes, PI-3,5-P_2_ exists in late compartments of the endosomal pathway, and PI-4-P is enriched at the Golgi complex but also present at the plasma membrane[41]. More studies have focused on the function of PI-4,5-P_2_ rather than that of PIP_3_ as the steady-state abundance of PIP_3_ at the plasma membrane is low. However, local levels of PIP_3_ change dynamically following stimulation [7]. Indeed, αSyn has been shown to form discrete foci at the cellular plasma membrane, whereby the abundance and localization of these foci correlate with pools of PIP_3_ and PI-4,5-P_2_ [25].

Expanding on these previous findings, we demonstrated that increased cellular PIP_3_ levels resulted in the formation of αSyn inclusions in an overexpression cultured cell line. Following cellular uptake, Bodipy-FL-PIP_3_ can be observed in small round vesicular structures resembling endosomes/lysosomes. PIP_3_ localization at endosomes has been previously reported [17]. The current study confirmed that the PIP_3_- and αSyn-positive inclusions were localized in the lysosomes, suggesting that this could be the site of their encounter. We speculate that the association with PIP_3_ in lysosomes initiates αSyn aggregation, which leads to disruption of the lysosomal membrane and, in turn, cascading aggregation of cytoplasmic αSyn [18, 49]. Importantly, the lysosomal localization of these inclusions closely resembles the pathological feature of LB as it has been reported that lysosome-like vesicles could be detected inside or at the edge of LB inclusions [33, 51]. We also present evidence that PIP_3_ hastened the formation of aggregates from endogenously expressed αSyn using primary neurons. In primary cells, PIP_3_ and αSyn aggregates were predominantly localized in the presynaptic region in the neuronal processes. Presynaptic localization PIP_3_ observed here is consistent with a previous study that reported developing cortical neurons exhibited intense PIP_3_ levels in their axon and growth cone during the period of rapid axon growth [38]. Our result also agrees with the previous studies using mouse models showing αSyn being enriched in the presynaptic region [11] and phosphorylation of endogenous αSyn initiates at the presynaptic region and spreads through the axon to the cell body [5]. Intriguingly, the phenotype observed in the neuronal processes of primary neurons fittingly recapitulates the initial stage of the formation of Lewy neurites found in diseased synucleinopathy brains.

A more thorough assessment on how distinct PIPs affect αSyn aggregation was performed using in vitro-based assays. In vitro protein–lipid interaction studies and seeding assays revealed that mono-, di-, and triphosphorylated PIPs bind to αSyn and promote their aggregation to different extents, with PIP_3_ ranked top on both assessments. The higher affinity of PIP_3_ for αSyn than of other PIPs could be attributed to the stereochemistry of the phosphorylation of the inositol moiety. The results of PIPs–αSyn binding indicated that single phosphorylation of PI promotes the interaction with αSyn, probably due to electrostatic contacts between the N-terminal domain of αSyn and the negative phosphate group. Among the mono-phosphorylated PIPs, PI-3-P showed the highest affinity for αSyn, which increased if a second phosphate group was added at position 5 or was affected if the position was 4. Considering the stereochemistry of the inositol moiety, a double phosphorylation at positions 3 and 5 would enhance the interaction because both phosphate groups are oriented in the same direction. Alternately, if double phosphorylation takes place at positions 3 and 4, the phosphate groups are oriented in different directions, negatively affecting the interaction. Finally, PIP_3_ showed the strongest interaction, probably due to the additive effect of simultaneous phosphorylation at positions 3, 4, and 5, while maintaining favorable stereochemistry.

This is the first detailed report showing a direct association between PIP_3_ and αSyn which is attributed to specific interaction with the N-terminal and NAC regions of αSyn, as revealed by NMR spectra. Collectively, in vitro experiments help clarify the mechanism of how PIP_3_ initiates αSyn misfolding. Notably, the interaction with PIP_3_ induces the formation of fibrils exhibiting structural and biochemical properties similar to those amplified from PD brains. Recent studies have evaluated the relationship between structural polymorphism and disease diversity to understand the mechanisms by which a single amyloidogenic protein causes different diseases. For example, αSyn causes both PD and multiple system atrophy (MSA). Ultrastructural analysis of αSyn amyloid-like fibrils extracted from patients’ brains showed that brain αSyn fibrils differ between those in MSA and in PD/DLB, with the former being predominantly twisted and the latter being mostly straight rod-like [13, 48, 55]. The current study demonstrated that the PIP_3_-derived fibrils showed rod-like morphology similar to that of the PD brain-derived fibrils. To the best of our knowledge, this is also the first study to connect the accumulation of a specific lipid to the creation of fibrils with morphological and biochemical similarities to those of PD-related fibrils.

Previous studies on the interplay between αSyn and other lipids were mainly based on in vitro and in vivo preclinical studies with little direct investigation of PD brain samples [19]. The key strengths of this study are that we provide evidence showing PIP_3_ induces the pathological aggregation of αSyn to form fibrils showing structural and biochemical resemblance to those derived from PD brains and, importantly, the accumulation of PIP_3_ colocalized with pSyn in postmortem PD brain samples. Regarding the expression levels of SYNJ1, we did not observe a significant increase in the PD brains of our study population, indicating that PIP_3_ accumulation is a common pathological change in both familial (PARK20) and sporadic PD and the reduced SYNJ1 activity is not the sole reason for the accumulation of PIP_3_. It would be an important theme to investigate the prevalence of SYNJ1 deficiency among the sporadic PD patients and to explore the upstream causative mechanism of PIP_3_ accumulation in the remaining populations in future studies. We also acknowledge the limitation of the small sample size of postmortem cohorts used in our study which may not sufficiently reflect the heterogeneity of PD. Larger sample size may be required in future studies.

In conclusion, aberrant interaction of αSyn with PIP_3_ which accumulates upon the loss of function of lipid phosphatase SYNJ1, promotes the transition of physiological αSyn to pathological assemblies showing structural and biochemical similarities to those derived from PD brains. This study thereby highlights an emerging role of PIP_3_ in the context of PD pathogenesis and opens new therapeutic perspectives targeting PIP_3_ to improve PD pathology.

## Supplementary Information

Below is the link to the electronic supplementary material.

Supplementary file1 (PDF 4861 kb)

## Data Availability

The data generated and analyzed for this manuscript are available from the corresponding authors upon reasonable request.
