# Supplementary material for: Phosphatidylinositol-3,4,5-trisphosphate interacts with alpha-synuclein and initiates its aggregation and formation of Parkinson’s disease-related fibril polymorphism
Source: Acta Neuropathol. 2023 Mar 20;145(5):573–95. doi: 10.1007/s00401-023-02555-3 (PMC10119223; doi:10.1007/s00401-023-02555-3)
Supplement: Supplementary file 1 — Supplementary file1 (PDF 4861 kb) [file 401_2023_2555_MOESM1_ESM.pdf]

Phosphatidylinositol-3,4,5-trisphosphate interacts with alpha-synuclein and initiates its aggregation and formation of Parkinson's disease-related fibril polymorphism

Chi-Jing Choong[1,a], César Aguirre[1,a], Keita Kakuda[1,a], Goichi Beck[a], Hiroki Nakanishi[b], Yasuyoshi Kimura[a], Shuichi Shimma[c], Kei Nabekura[a], Makoto Hideshima[a], Junko Doi[a], Keiichi Yamaguchi[d], Kichitaro Nakajima[d], Tomoya Wadayama[a], Hideki Hayakawa[a], Kousuke Baba[a], Kotaro Ogawa[a], Toshihide Takeuchi[e], Shaymaa Mohamed Mohamed Badawy[a,f], Shigeo Murayama[g], Seiichi Nagano[a], Yuji Goto[d], Yohei Miyanoiri[h], Yoshitaka Nagai[e], Hideki Mochizuki[†,a,*], and Kensuke Ikenaka[†,a,*]

[†]Equal correspondence

[1]Equal contribution

[a] Department of Neurology, Osaka University Graduate School of Medicine, 2-2 Yamadaoka, Suita, Osaka 565-0871, Japan

[b] Lipidome Lab Co., Ltd, Akita-city, Akita 010-0825, Japan.

[c] Department of Biotechnology, Graduate School of Engineering, Osaka University, 2-1 Yamadaoka, Suita, Osaka 5650871, Japan.

[d] Global Center for Medical Engineering and Informatics, Osaka University, Japan, 2-1 Yamadaoka, Suita, Osaka 565-0871, Japan

[e] Department of Neurology, Kindai University, 3-4-1 Kowakae, Higashiosaka City, Osaka 577-8502, Japan

[f] Department of Agricultural Biochemistry, Faculty of Agriculture, Zagazig University, Zagazig, Egypt.

[g] Brain Bank for Neurodevelopmental, Neurological and Psychiatric Disorders, United Graduate School of Child Development, Osaka University, 2-2, Yamadaoka, Suita, Osaka, 565-0871, Japan.

[h] Institute for Protein Research, Osaka University, 3-2 Yamadaoka, Suita, Osaka 565-0871, Japan

* Kensuke Ikenaka, Hideki Mochizuki

Email: ikenaka@neurol.med.osaka-u.ac.jp

Postal address: Department of Neurology, Yamadaoka 2-2, Suita, Osaka 565-0871, Japan.

Tel: +81-06-6879 3571; Fax: +81-06-6879 3579.

Supplementary figures

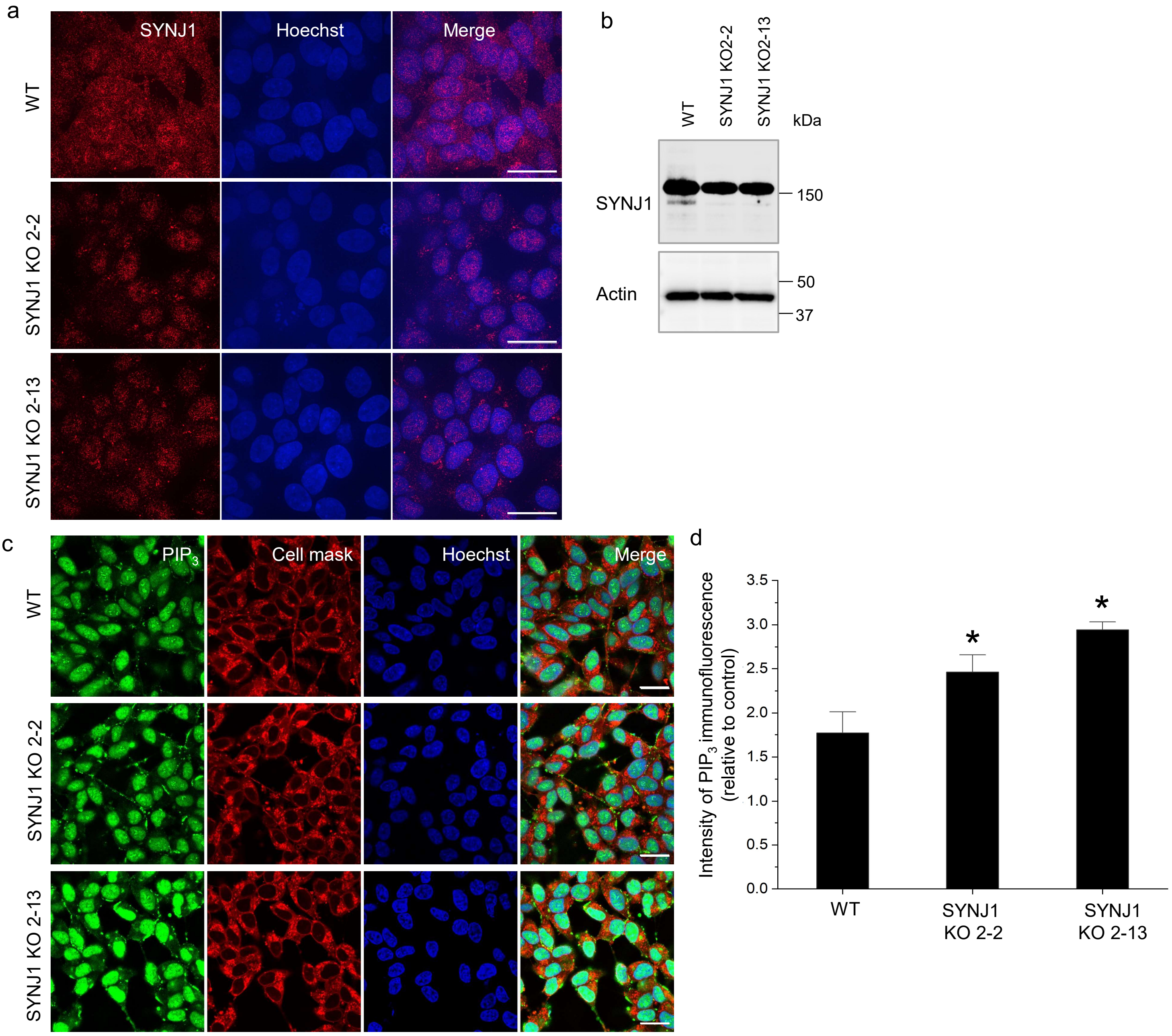

Supplementary Fig. 1 Characterization of SH-SY5Y SYNJ1 knockout clones. (**a**) Verification of SYNJ1 knockout clones by immunofluorescence staining (Scale bar = 20 μm) and (**b**) immunoblot analysis. (**c**) Immunofluorescence staining of PIP_3 in control SH-SY5Y and SYNJ1 knockout clones. Scale bar = 20 μm. (**d**) The intensity of PIP_3 immunofluorescence was significantly increased in the cytoplasmic region of SYNJ1 KO clones. Data are presented as the mean ± SEM ($n = 3$); one-way ANOVA followed by Dunnett's post hoc test compared to control; $*P < 0.05$.

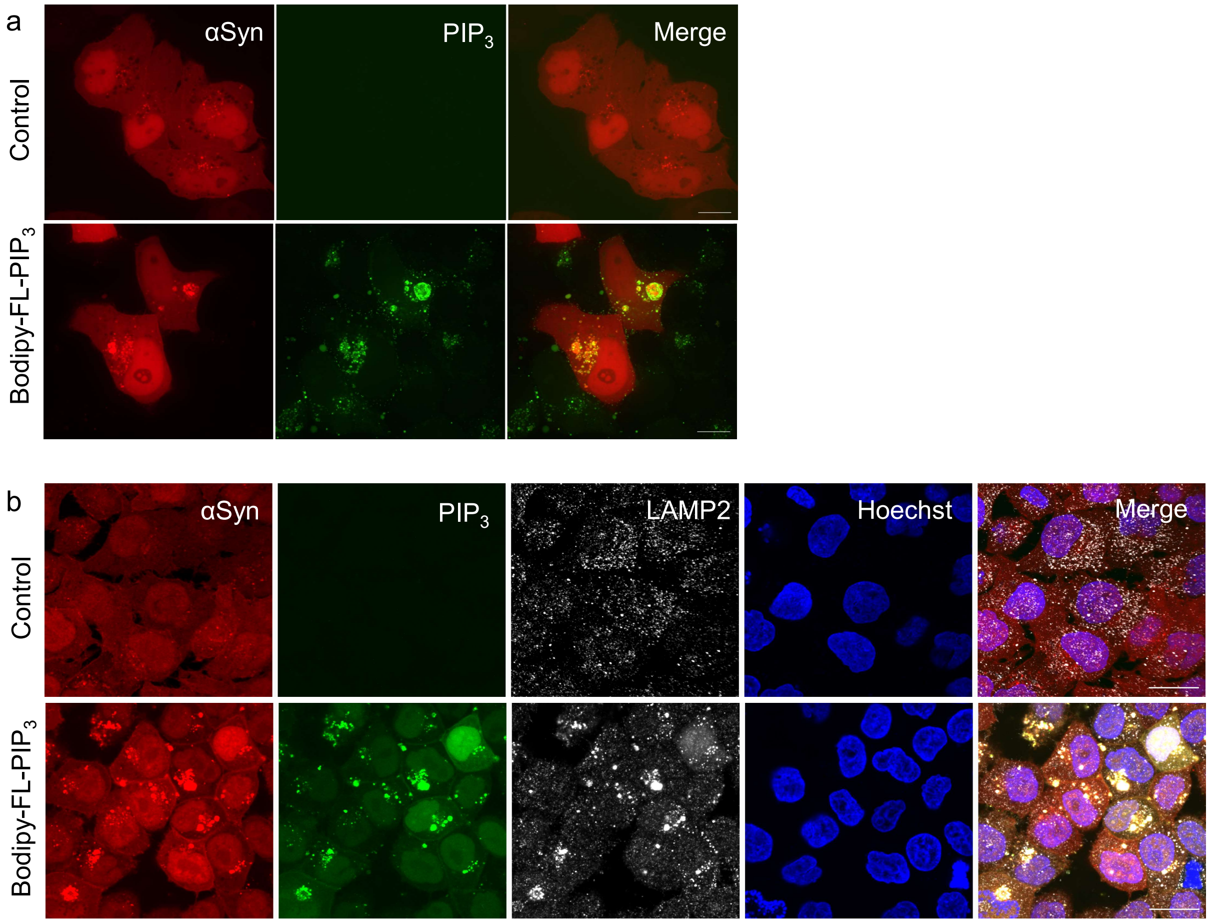

Supplementary Fig. 2 (**a**) Delivery of PIP_3 into Hela cells transiently overexpressing αSyn. Representative confocal images of Hela transiently overexpressing αSyn mRFP (red) treated with BODIPY-PIP_3 (green) using histone carrier. Scale bar = 10 µm. (**b**) Staining of control and Bodipy-FL®PIP_3 (green)-treated Hela-αSyn mRFP (red) cells with lysosomal marker LAMP2 (grey). Cells are counterstained for nuclei with Hoechst (blue). Scale bar = 20 µm.

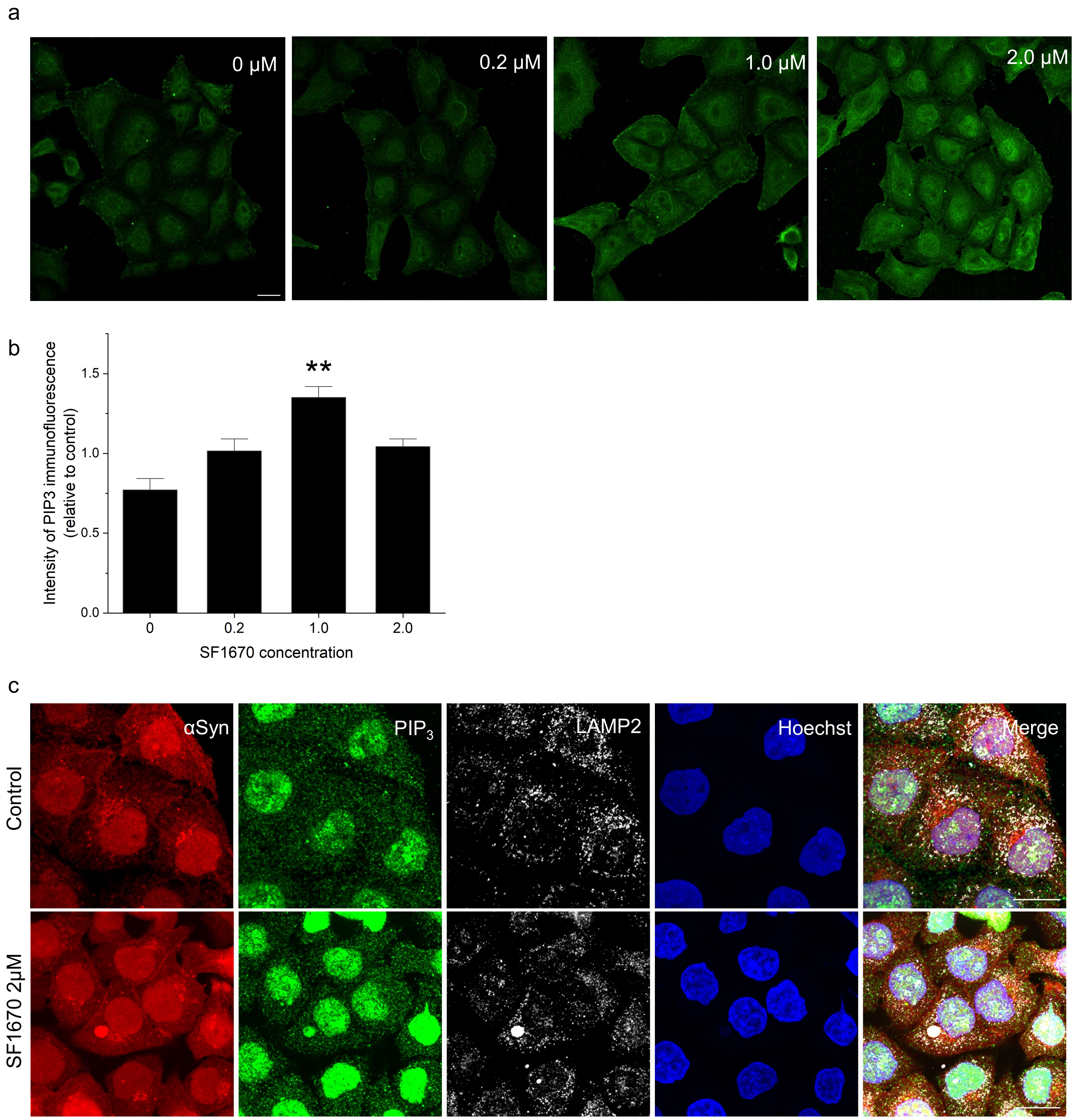

Supplementary Fig. 3 SF1670-induced upregulation of cellular PIP_3 level. (**a**) Representative images showing immunofluorescence of PIP_3 with each drug concentration. Scale bar = 10 μm. (**b**) Intensity of PIP_3 immunofluorescence was significantly increased following SF1670 treatment. Data are presented as the mean ± SEM (n = 3); one-way ANOVA followed by Dunnett's post hoc test compared to control; **$P < 0.01$. (**c**) Double immunofluorescence staining of PIP_3 (green) and LAMP2 (grey) in control and SF1670-treated HeLa-αSyn-mRFP cells (red). Cells are counterstained for nuclei with Hoechst (blue). Scale bar = 20 μm.

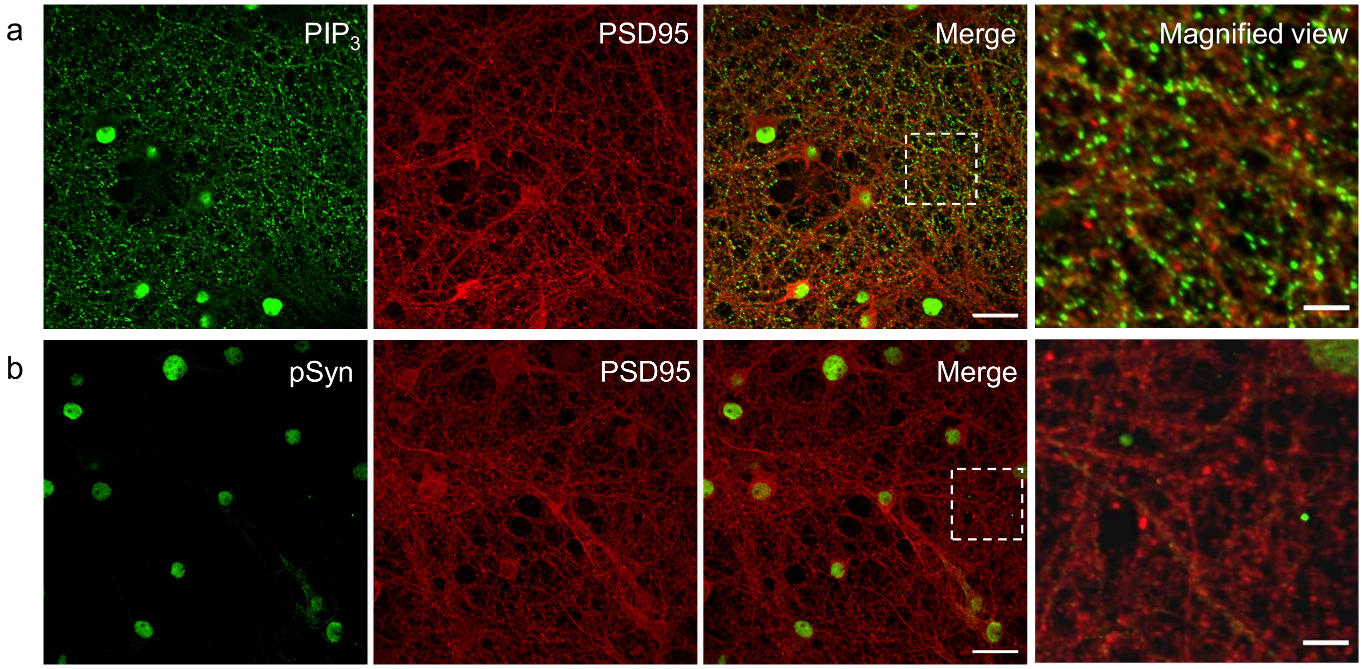

Supplementary Fig. 4 Assessment of postsynaptic localization of (**a**) PIP3 and (**b**) pSyn (green) by co-staining with postsynaptic marker PSD95 (red). Scale bar = 10 µm. Magnified views of dashed boxed area are shown in the right panel. Scale bar = 5 µm.

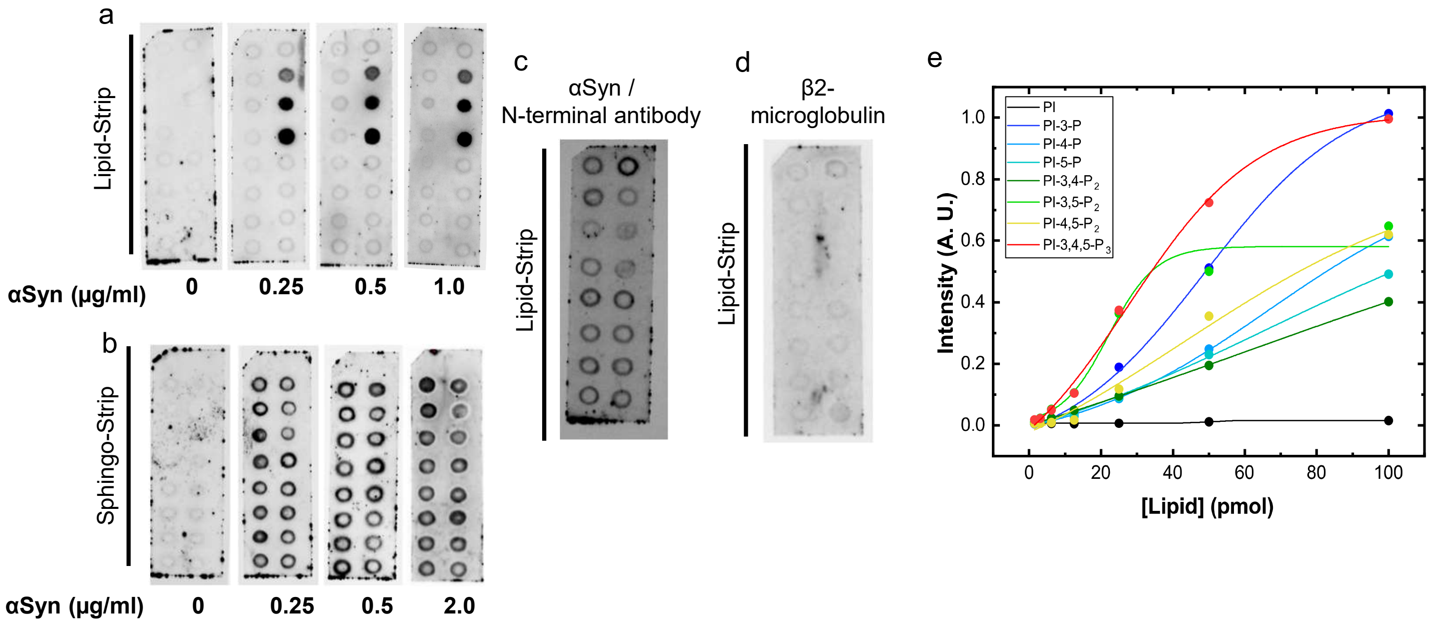

Supplementary Fig. 5 Lipid-binding profile of different proteins to lipid strip. (**a, b**) Four different concentrations of αSyn are evaluated for (**a**) lipid strip and (**b**) Sphingo strip. (**c**) Lipid-strip incubated with 1.0 µg/mL of αSyn and detected using a monoclonal antibody F-11 recognizing the N-terminus of αSyn. (**d**) Lipid-strip incubated with β2-macroglobulin at the concentration of 1.0 µg/mL. (**e**) Representative example of the fitting of the experimental intensities obtained from the PIP array (dots) to the Boltzmann equation model (lines) to determine the parameter of binding $[Lipid]_{50\%}$.

a

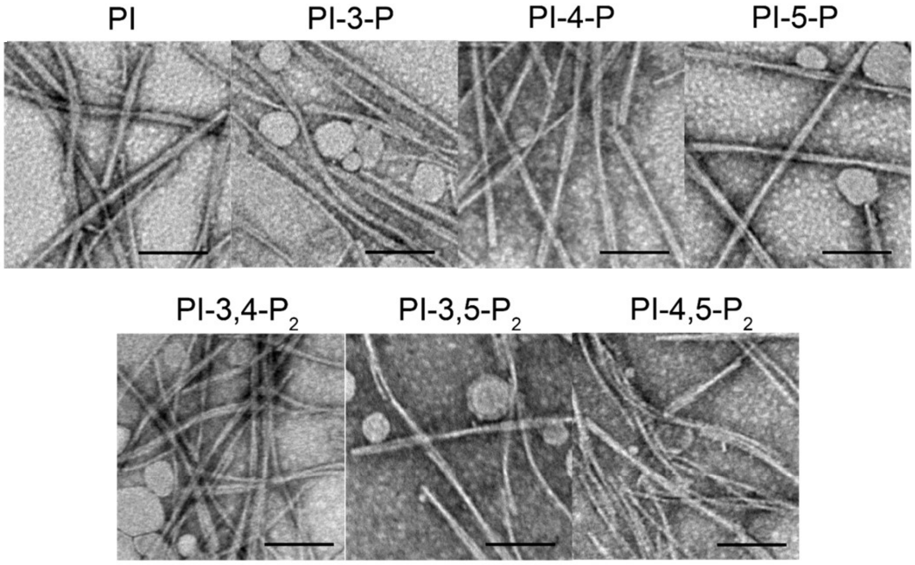

b

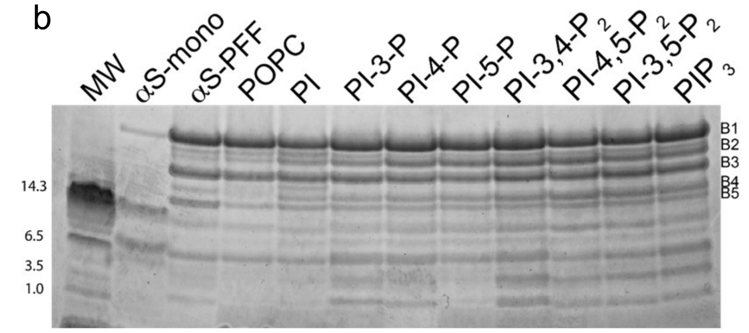

Supplementary Fig. 6 Conformation and biochemical characteristics of αSyn fibrils formed in the presence of POPC-phosphatidylinositol-derived vesicles. (**a**) TEM visualization of αSyn fibrils formed in the presence of phosphatidylinositol (PI) and mono- and di-phosphorylated phosphatidylinositol. (**b**) Proteinase K resistance assay of αSyn monomers (αS-mono) and preformed fibrils (αS-PFF), followed by the fibrils obtained in panel A. Bands numbered from B1 to B5 are employed for analysis and comparison.

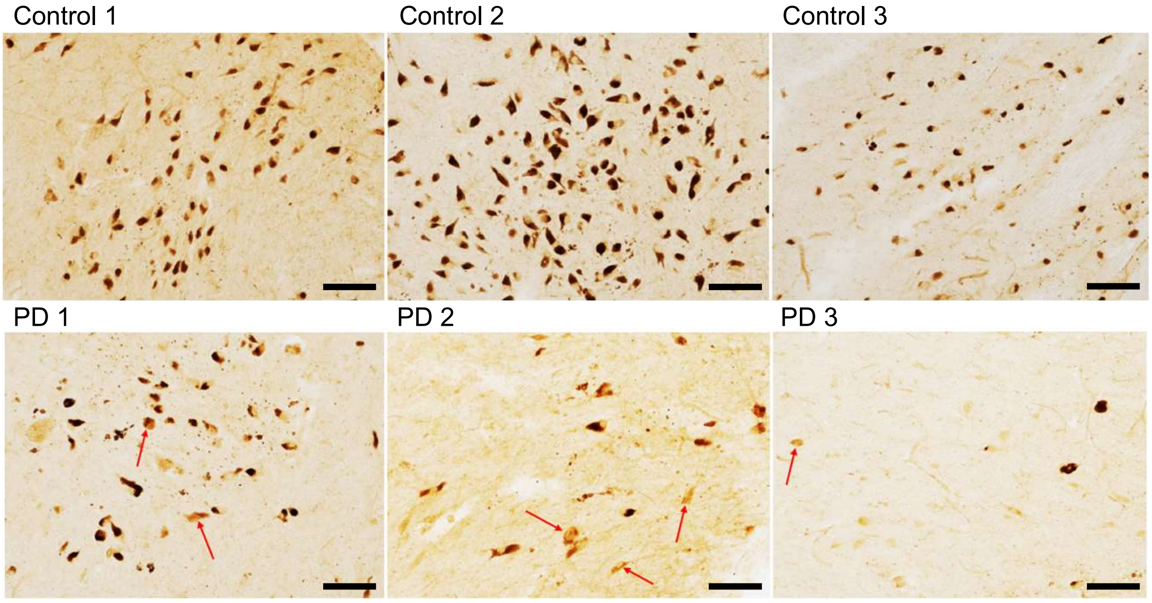

Supplementary Fig. 7 Immunohistochemical staining of PIP_3 (without Nissl counterstaining) in the substantia nigra of control and PD patients (*n* = 3 each). The dark brown cells are neuromelanin-containing cells. PIP_3 accumulations are highlighted by red arrows. Scale bar = 100 μm.

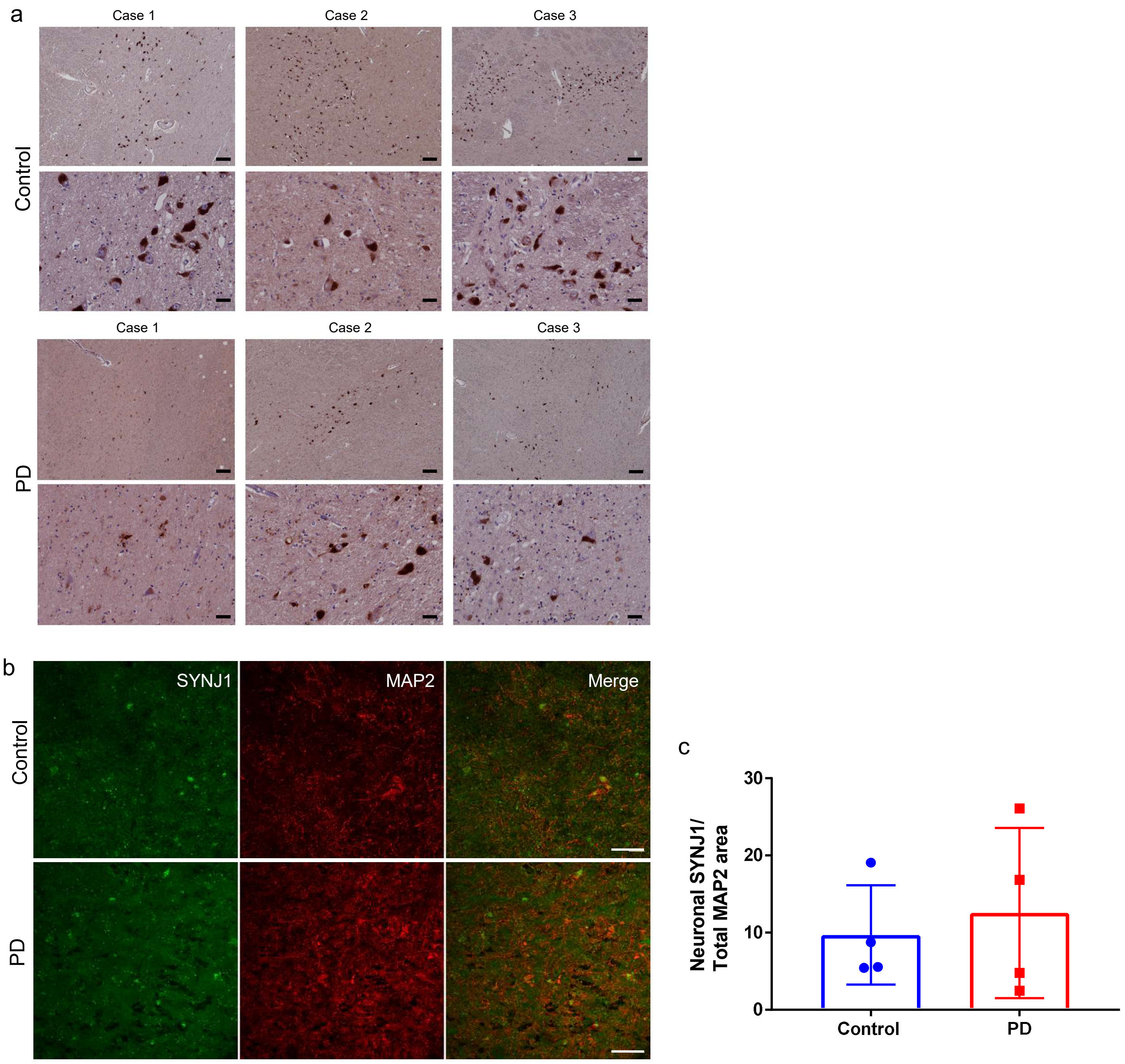

Supplementary Fig. 8 Analysis of SYNJ1 level in the brain samples of control and PD patients. (**a**) Immunohistochemical staining of SYNJ1 with Nissl counterstaining in the substantia nigra of control and PD patients (n = 3 for control; n = 3 for PD). The dark brown cells are neuromelanin-containing cells. Scale bar = 100 µm for low magnification images and 25 µm for high magnification images. (**b**) Double immunofluorescence staining of SYNJ1 and MAP2 in the brain samples of control and PD patients. Scale bar = 50 µm. (**c**) Quantitative analysis of neuronal SYNJ1 intensity per unit area of MAP2. Data are expressed as the mean ± SEM (n = 4 for control; n = 4 for PD).
